# Durably Superhydrophobic Magnetic Cobalt Ferrites for Highly Efficient Oil–Water Separation and Fast Microplastic Removal

**DOI:** 10.1021/acs.langmuir.4c02420

**Published:** 2024-10-07

**Authors:** Anhua Ren, Oriol Rius-Ayra, Min Kang, Nuria Llorca-Isern

**Affiliations:** † College of Engineering, Nanjing Agricultural University, No. 40 Dianjiangtai Road, Nanjing 210031, China; ‡ CPCM Departament de Ciència dels Materials i Química Física, Facultat de Química, Universitat de Barcelona, Martí i Franquès 1 - 11, 08028 Barcelona, Spain

## Abstract

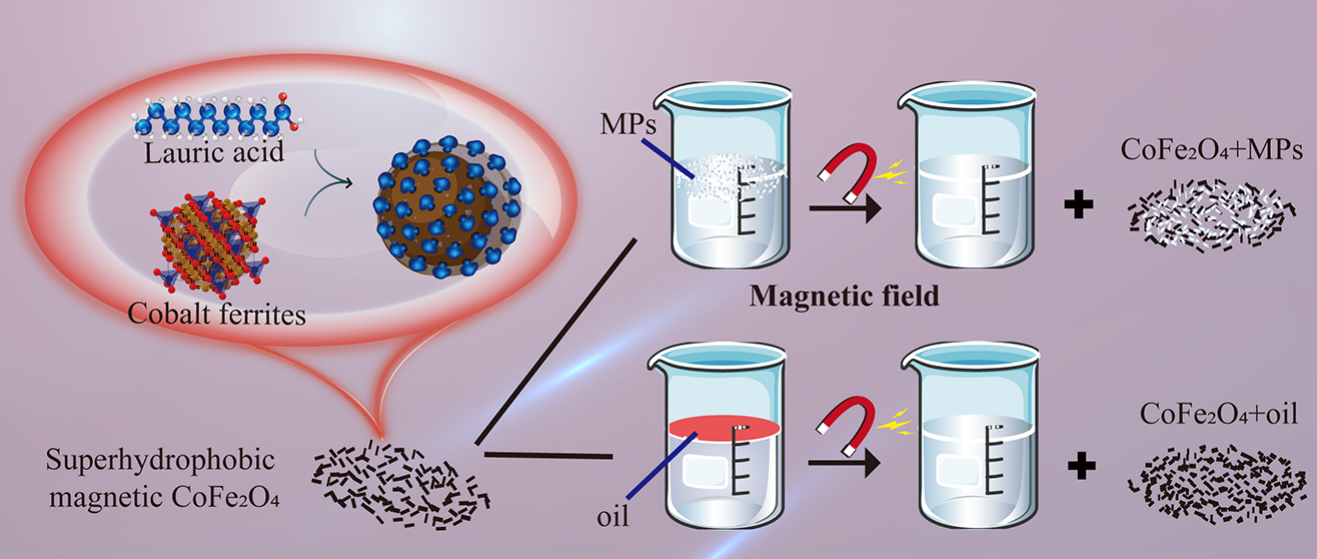

Microplastic pollution has become a primary global concern in the 21st century. Recyclable magnetic particles with micro-nanostructures are considered an efficient and economical way to remove microplastics from water. In this study, superhydrophobic magnetic cobalt ferrite particles were prepared by using a simple coprecipitation method combined with surface functionalization. The micromorphology, chemical composition, hysteresis loop, and surface contact angle of the functionalized cobalt ferrite were characterized. The separation efficiency and absorption capacity of cobalt ferrite particles in water–oil separation and microplastic removal were investigated. The results showed that the saturation magnetic field intensity of cobalt ferrite was 65.52 emu/g, the residual magnetization intensity (Mr) was 18.79 emu/g, and the low coercivity was 799.83 Oe. Cobalt ferrites had stable superhydrophobicity in the pH range of 1–13. The separation efficiency of cobalt ferrite powder for four oil–water mixture separations was higher than 94.2%. The separation efficiency was as high as 99.6% in the separation of the hexane and water mixtures. Due to the synergistic effect of the hydrophobic effect and van der Waals force, the functionalized magnetic cobalt ferrite had a high and stable microplastic removal efficiency and capture capacity. The removal efficiency of microplastics was close to 100%, and the capture capacity was 2.56 g/g. After ten microplastic removal cycles, the removal efficiency reached more than 98%, and the surface contact angle was still greater than 150°.

## Introduction

The rapid development of industries around the world today has also brought about many environmental pollution problems. Microplastic particles (MPs) are considered as a new environmental pollutant in the 21st century.^1,2^ Plastic fragments with a diameter of less than 5 mm, defined as microplastics, are considered an emerging environmental pollutant that has received widespread attention due to their potential adverse effects on organisms.^3,4^ Due to the relatively large specific surface area of MPs, heavy metals and persistent organic pollutants (POPs) tend to adhere and accumulate on MP surfaces and then migrate in the environment.^5^ It is predicted that the total mass of plastic debris accumulated in the ocean may increase to 250 million metric tons (t) by 2025. Microplastics have been readily absorbed by marine organisms and enter the food chain, thereby jeopardizing public health.^6^ People have been exposed to microplastics in food and drinking water. Long-term exposure to MPs can cause chronic toxicity, including impaired reproduction and malnutrition, posing a threat to biota and humans.^7^ Both the serious threats and potential hazards posed by microplastics highlight the need to separate them from the environment. In recent years, various methods and techniques have been used to remove microplastics. Various strategies have been introduced in real life to remove microplastics such as multistage removal in wastewater plants,^8^ membrane filtration,^9^ adsorption,^10^ coalescence and agglomeration,^11^ biodegradation,^12^ photocatalysis,^13^ and so on. Many aspects, such as cost, safety, flexibility, sustainability, and separation efficiency, are considered. The above strategies for removing microplastics have the shortcomings of being a time-consuming process, complicated operation, high cost, and difficulty in meeting the needs of large-scale production. Meanwhile, in real life, the water environment often faces more complex problems, including organic matter and oil pollution.^14^

In recent years, the use of magnetic materials as an alternative to filtration or multistage processes has created an urgent need for new recyclable and efficient methods of removing microplastics. Treatment to remove microplastics and other contaminants from water is of great interest. Magnetic materials have the advantage of being renewable and reusable, minimizing the generation of hazardous waste. Recent studies have shown encouraging results. Misra et al.^15^ used magnetic Fe_2_O_3_/SiO_2_ core–shell nanoparticles functionalized with polymetallic oxonate ionic liquids for microplastic removal. Herrmann et al.^16^ removed multiple pollutants from water by metal oxonate loaded ionic liquid phase (POM-SIRP). Guo et al.^17^ created a novel core–shell magnetic biosorbent. Rius-Ayra et al.^18^ prepared superhydrophobic nanostructured CuFeCo powder alloys for the capture of microplastics. Mayorga-Burrezo^13^ et al. created an Fe_3_O_4_@BiVO_4_ microrobot for the removal of microplastics and dyes in the presence of a mixture of photocatalytic and transversely rotating magnetic fields. These particles also show great potential for the removal of oil layers from water. Cobalt ferrite (CoFe_2_O_4_) is an interesting material with high magnetocrystalline anisotropy, high coercivity, and moderate magnetization strength,^19^ Nano spinel ferrite has attracted attention due to its small particle size, narrow particle size distribution, and large surface area.^20^ Several methods have been developed to synthesize cobalt ferrites, including hydrothermal,^21^ sol–gel,^22^ chemical reduction,^23^ microwave synthesis,^24^ mechanical alloying,^25^ and coprecipitation.^26,27^ Among these methods, the coprecipitation method is characterized by low toxicity, rapid preparation, and low cost.

In recent years, superhydrophobic materials have proven to be an innovative strategy for removing pollutants from water and have been widely studied and applied in the fields of anticorrosion,^28^ antifogging,^29^ self-cleaning,^29^ and antifouling.^30,31^ Generally, a material surface is defined as superhydrophobic when its water contact angle is greater than 150°, its sliding angle is less than 10°, or its contact angle hysteresis is less than 10°.^32,33^ Studies have shown that the microplastic removal efficiency is related to surface wettability and wetting stability. Materials with rough structure and low surface energy have strong water repellency and oil absorption capacity and show high efficiency in treating oily wastewater pollution.^34^ In order to achieve long-lasting separation efficiency and separation performance, superhydrophobicity should be achieved by increasing surface roughness and reducing surface energy.^35^ Therefore, there is an urgent need to investigate functionalization using magnetite MNPs. Saturated fatty acids have the property to regulate wettability by modulating surface groups. Low-cost lauric acid, a low surface energy fatty acid, dehydrates with the surface hydroxyl groups of the nanomaterials, and long carbon chains are distributed on the surface of the nanomaterials to reduce their surface energy in order to achieve superhydrophobic properties.^36^ Huang et al.^37^ used lauric acid modified superhydrophobic cellulose composite membranes for oil/water separation. However, the functionalization of magnetic nanoparticles by lauric acid modification for contaminant removal, such as microplastics, has rarely been studied. In addition, despite a large number of studies on microplastic removal, most of them focus on the removal efficiency, and few analyze the mechanism of microplastic removal in depth.

Here, we report an efficient alternative purification approach to remove microplastics and organic contaminants from water using magnetically functionalized cobalt ferrite particles. The study was based on a coprecipitation method and surface modification, where lauric acid adsorbs on the surface of cobalt ferrites to produce cobalt ferrite particles with stabilized hydrophobicity and magnetic properties. The cobalt ferrites can be recycled by using permanent magnets. In this study, we evaluated the ability of functionalized magnetic cobalt ferrites to remove microplastics and their potential in purifying water resources. We also investigated the trapping mechanism between cobalt ferrites and microplastics. In addition, we highlight the potential application of functionalized magnetic cobalt ferrites in water–oil separation.

## Experimental Section

### Synthesis of the Cobalt Ferrites

**Figure 1:**
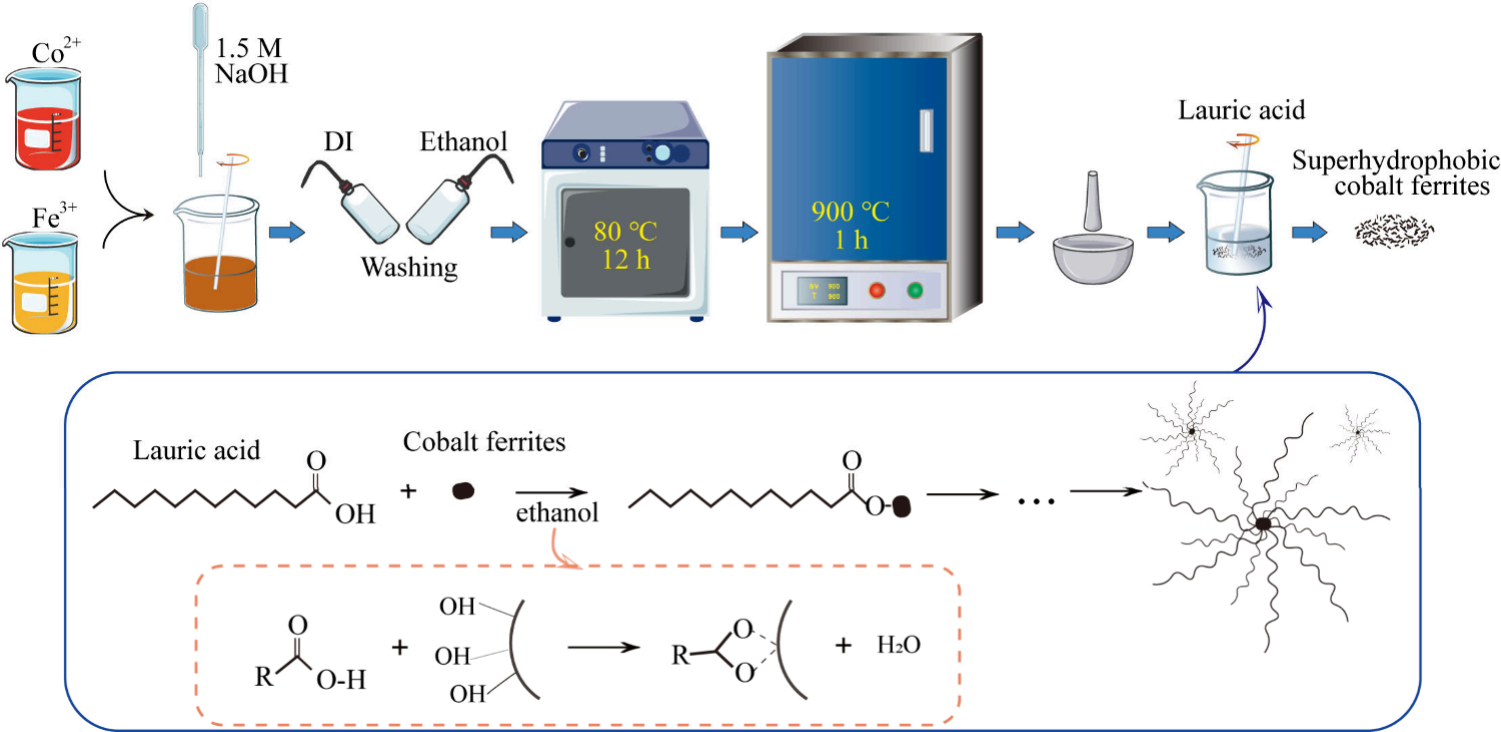
Procedure diagram of superhydrophobic cobalt ferrite preparation.

Two 25 mL solutions of 0.4 M FeCl_3_·6H_2_O (Scharlau, AR) and 0.2 M CoCl_2_·6H_2_O (Scharlau, AR) in deionized water were prepared separately until the salts were completely dissolved. Once they were dissolved, they were mixed and 2 mL of octanoic acid (Scharlau, AR) was added drop by drop with vigorous and constant stirring. Next, a 1.5 M NaOH (Scharlau, AR) solution was added; the addition was carried out slowly until the pH value (pH = 8) desired was reached. The solution obtained was heated at 80 °C with stirring for 1 h, and the product obtained was allowed to cool to room temperature. The main byproduct of the reaction was rinsed three times with deionized water and centrifuged at 1500 rpm for 2 min. The obtained solid was left after rinsing in the oven at 80 °C for 8 h to completely dry. Finally, a heat treatment was carried out at 900 °C for 1 h. Figure 1 reveals the procedure for fabrication of cobalt ferrites.

### Surface Modification

0.5 g of lauric acid (C_12_H_24_O_2_, Scharlau, AR) was dissolved in 5 mL of absolute ethanol. The cobalt ferrites were added to this solution and left for 24 h at room temperature, preferably with a magnet under the vial to increase the surface area. After 24 h, the lauric acid solution was removed with the help of a magnet and the ferrites were rinsed with absolute ethanol several times. Finally, ferrites were left to dry in the air.

### Characterization

A JEOL J-7100 field emission scanning electron microscope (FE-SEM) coupled to an energy-dispersive spectroscopy (EDS) detector was used to assess the morphology, semiquantitative composition, and element distribution of the powder as well as to study the sample surfaces. X-ray diffraction (PANalytical X’Pert PRO MPD, with radiation Fe filtered Co K with a wavelength of 1.79 Å) was utilized to examine the phase structures of cobalt ferrites with surface modification. The Scherrer equation^38^ is as follows:
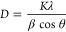
1*D* presents the average size of the cobalt ferrite grain size; the value of *K* approximates the dimensionless shape factor, which has a value close to 1; λ presents the wavelength of the X-ray; β is the line broadening at half the maximum intensity (fwhm), after subtracting the instrumental line broadening, in radians. θ presents the Bragg angle.

Additionally, high-resolution X-ray photoelectron spectroscopy (HR-XPS) was used on a PHI ESCA-5500 using a monochromatic X-ray source (Kα(Al) = 1486.6 eV and 350 W) and a Multipak (9.8) was used for the deconvolution analysis. Variations in the magnetic parameters were measured at 300 K using an MPMS XL superconducting quantum interference device (SQUID) magnetometer from Quantum Design.

### Contact Angle Measurements

Contact angle measurements were performed in an open atmosphere at room temperature. To avoid the roughness effect, cobalt ferrite powder was sprinkled on a glass slide coated with double-sided tape and flattened using another glass slide, and excess powder was removed.

First, deionized water (DI) and oil (hexane) were used to measure the water contact angle (WCA) and oil contact angle (OCA) of cobalt ferrites, respectively. Then, HCl and NaOH (Scharlau, AR) were used to prepare solutions with different pH values (1, 3, 5, 7, 9, 11, and 13). The surface WCA and sliding angle (SA) of cobalt ferrites were tested by using these solutions.

During the experiment, 3 μL of deionized water or oil (hexane) was dropped onto the sample surface. The static WCA and OCA of cobalt ferrite powder were captured using a Levenhuk DTX digital microscope. To measure the SA, a 3 μL droplet of deionized water was similarly placed on a cobalt ferrite powder-coated glass slide. The slide was then gradually tilted at a controlled rate, until the droplet began to slide. The angle at which the droplet started to slide was recorded as the SA. The captured droplet images were processed by using ImageJ 1.53a software. Each sample was measured at different locations at least five times to ensure data reliability and stability, and the average and standard deviations of these measurements were calculated. Statistical significance of the results was assessed by using ANOVA, and 95% confidence intervals were calculated for each measured parameter to provide an estimate of measurement precision.

### Oil–Water Separation

Based on the microplastic removal experiment, we also conducted a water–oil separation test to test the ability of cobalt ferrites to separate water and oil. Fill a glass bottle with 5 mL of water and then add 20 μL of oil dyed with oil red O (hexane, petroleum ether, xylene, olive oil). Add 10–32 mg of cobalt ferrites to each bottle. Shake the bottle by hand and wait for 10 min; then observe the liquid in the glass bottle. Place a hand-held magnet on one side of the glass bottle to absorb cobalt ferrite powder, tilt the bottle, pour out the oil-removed water, and measure the volume of the water. The powder was weighed before and after separation, and the adsorption capacity of cobalt ferrites was obtained according to eq 2.^39^ Calculate the separation efficiency of the powder according to eq 3.^39^ Finally, the water was poured out, while the powder was rinsed several times with ethanol and dried in a fume hood. To recover the CoFe_2_O_4_ particles so that they can be used again for oil removal, after each oil/water separation cycle, they were washed three times with absolute ethanol and then dried in a fume hood for a few minutes until the ethanol evaporated. Determination of residual oil in water and the presence of Oil Red O was performed by a Levonchuck DTX digital microscope
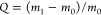
2where *m*_0_ is the mass of the cobalt ferrites before the oil/water separation and *m*_1_ is the mass of the cobalt ferrites after the oil/water separation
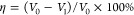
3where *V*_0_ is the volume of oil in oil/water mixtures and *V*_1_ is the volume of the oil lost after oil separation with superhydrophobic cobalt ferrites.

### Microplastics Removal

This research utilized high density polyethylene (HDPE) with a size of 99–167 μm to test the capture ability of cobalt ferrites. A 5 mL portion of water was placed in a glass bottle; then different amounts of microplastics were added and the solution was stirred and ultrasonically dispersed for a certain period. Since microplastics were superhydrophobic, they all floated on the water surface. Then a small amount of powders was put in, and under the action of a magnet, the powders were guided to capture the microplastics. Finally, a magnet was placed on one side of the glass bottle to absorb the magnetic powder, and then the glass bottle was tipped to drain out the liquid. The capture capacity of the powder was calculated according to eq 4. Through eq 5(40) the removal efficiency of microplastics was calculated
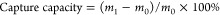
4where *m*_0_ is the mass of microplastics dropped in the water and *m*_1_ is the mass of the microplastics captured by cobalt ferrites in the beaker
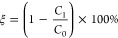
5where *C*_0_ is the concentration of microplastics dropped in the water and *C*_1_ is the concentration of the microplastics after being captured by cobalt ferrites in the beaker.

### Recycle Test

The recyclability of cobalt ferrite with functionalized magnetic properties was evaluated in ten cycles. The recyclability of cobalt ferrite is evaluated after microplastics are removed and after water–oil separation, respectively. In the case of water–oil separation, we conducted a recycling experiment using hexane as an example. After each step of the microplastic removal experiment and water–oil separation step, they were washed with ethanol and dried at 80°. The recovered pellets were then subjected to the next cycle.

## Results and Discussion

### Synthesis of Cobalt Ferrites

The proposed chemical reaction for preparing cobalt ferrites is as follows:
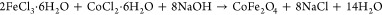
6

The reaction mechanism of cobalt ferrite particles modified by lauric acid ethanol solution is also shown in Figure 1. Some researchers have also introduced similar mechanisms.^41−44^ The carboxyl group of lauric acid absorbs a proton from ethanol, resulting in OH^2+^ leaving the group, which combines with ethanol to form water, while the remaining long chain part combines with the cobalt ferrite. Part of the carboxyl group of lauric acid loses a proton and shares an electron pair with ethanol after removing a hydroxyl group.

### Structure Analysis

**Figure 2:**
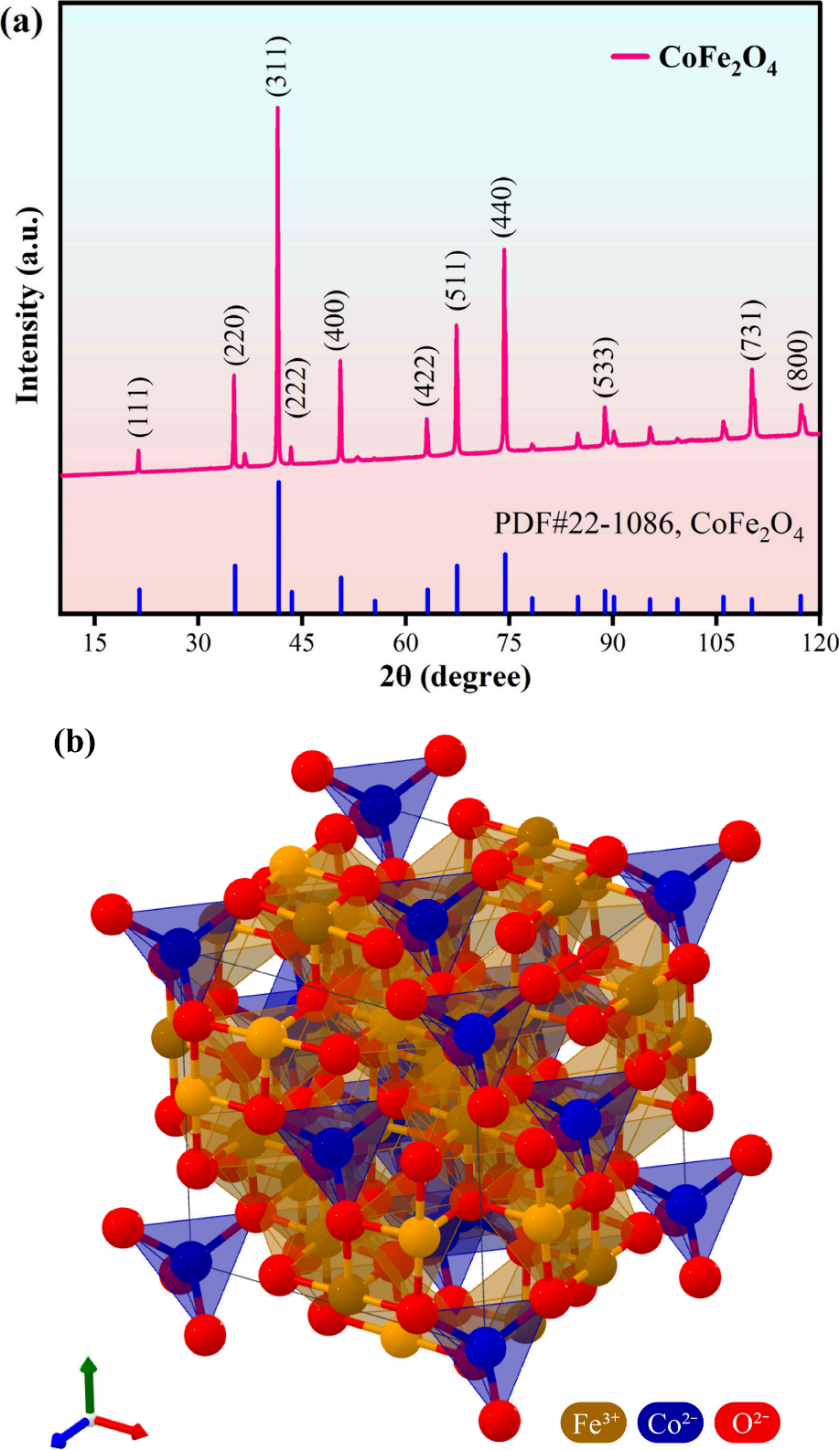
(a) XRD patterns of superhydrophobic cobalt ferrites. (b) Model of cobalt ferrites.

The XRD pattern was used to determine the formation and crystal structure of cobalt ferrites. As shown in Figure 2a, corresponding to the PDF#22-1086 card, it is thus clear that the product is a single phase of CoFe_2_O_4_. It can be seen that the XRD diffraction pattern contains diffraction peaks of (111), (220), (311), (400), (422), (511), (440), and (533), which is consistent with the anticubic spinel structure of the space group fd-3m (Figure 2b).^45^ Compared to standard PDF cards, the cobalt ferrites do not show significant changes in peak positions and relative intensities, indicating that surface functionalized cobalt ferrites do not alter the crystal structure of the powders. Taking the strongest diffraction peak (311) in XRD as the main peak, the average grain size of CoFe_2_O_4_ was obtained as 62.5 nm, calculated according to Scherrer’s formula 1.

**Figure 3:**
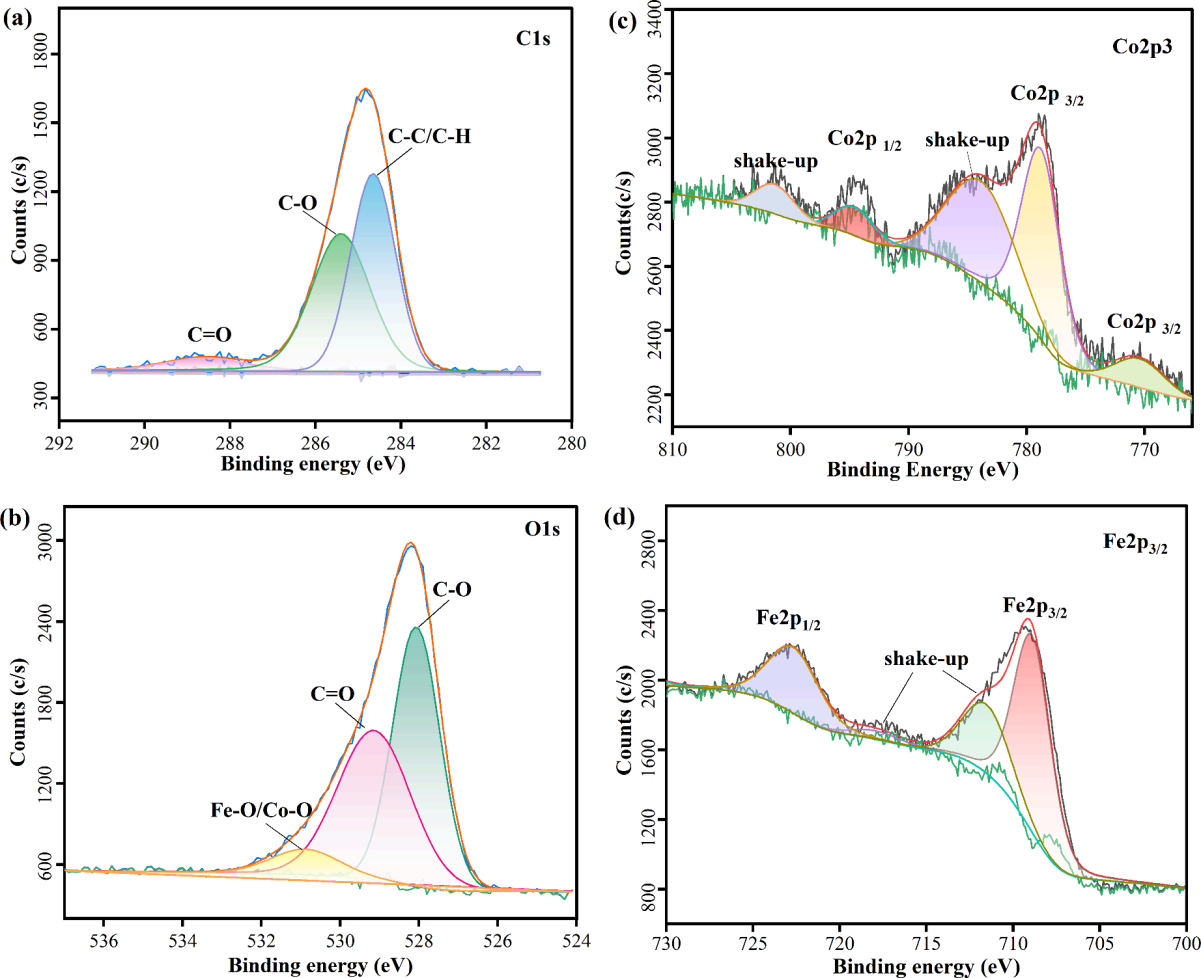
High-resolution XPS spectra of peaks (a) C 1s, (b) O 1s, (c) Co 2p, and (d) Fe 2p generated from the samples, respectively.

The overall stoichiometry and chemical valence of the elements of the prepared cobalt ferrites were analyzed by HR-XPS. Peaks of C 1s appeared at binding energies of about 284.67, 285.51, and 288.64 eV, respectively, corresponding to C—C/C—H, C—O, and C=O groups (Figure 3a).^46^ The C—O and C=O groups were attributed to the carboxylate functional group (—COOR) of lauric acid, indicating the successful modification of lauric acid on the surface of cobalt ferrite. In Figure 3b, the binding energy is about 528.07 eV, and peaks of O 1s appear at 529.14 eV and correspond to C=O and C—O, respectively.^47^ Meanwhile, the peak at the binding energy of about 530.89 eV is attributed to the lattice oxide Fe—O and Co–O features in CoFe_2_O_4_.^48,49^ The presence of Co 2p_3/2_ and its satellite peaks (shakeup) at binding energies of about 770.43, 778.80, and 783.84 eV, respectively, in Figure 3c, confirms the presence of the Co^2+^ state. The presence of Co^2+^ can be confirmed by the intensity of the Co 2p_3/2_ shakeup satellite since the low spin Co^3+^ cation only gives rise to much weaker satellite features than high spin Co^2+^ with unpaired valence 3d electron orbitals.^50^ In Figure 3d, the binding energies of about 708.90 and 711.53 eV correspond to Fe 2p_3/2_ and its satellite peak, which were used to determine the oxidation state of Fe as Fe^3+^. The binding energies of about 722.67 and 717.95 eV correspond to Fe 2p_1/2_ and its shock satellite peak (shakeup). By integrating the core level intensities, the atomic content ratio of Co to Fe elements can be obtained as 1:2.3 by the standard formula.^51^ Furthermore, the HR-XPS measurements agree with the XRD observations, confirming the formation of a single-phase cobalt ferrite rather than a mixed phase of CoO and Fe_2_O_3_.

### Morphological Observations

**Figure 4:**
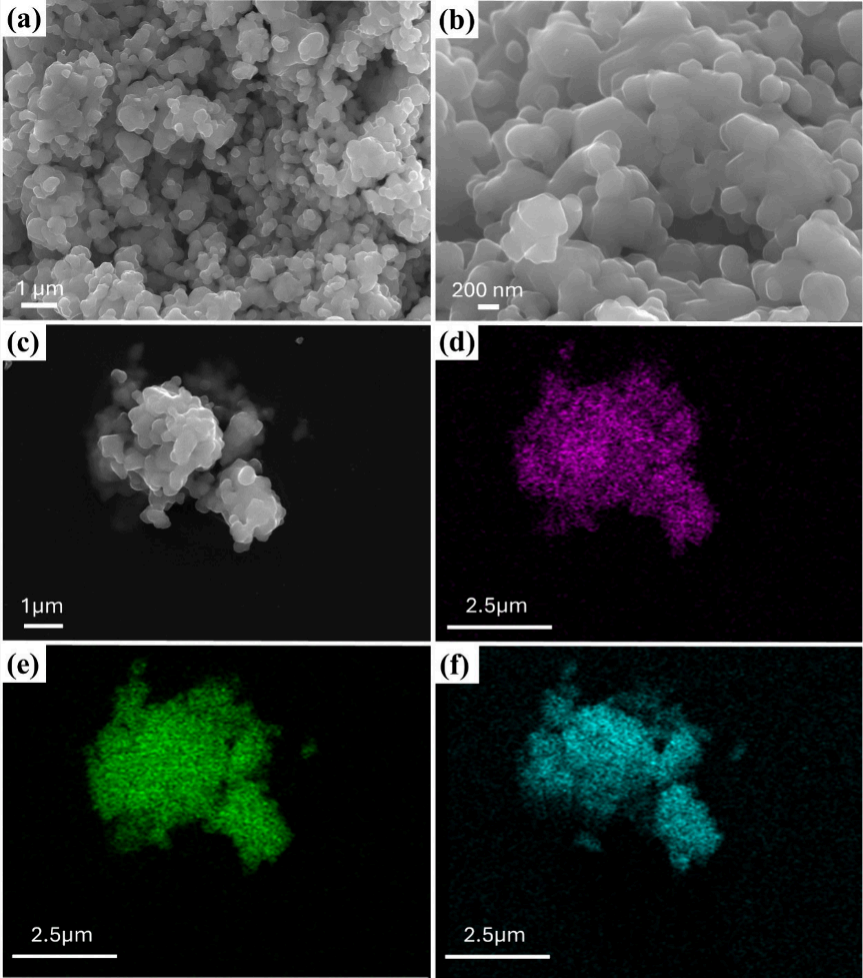
(a–c) FE-SEM of superhydrophobic cobalt ferrites; (d–f) EDS mapping of cobalt ferrites.

**Figure 5:**
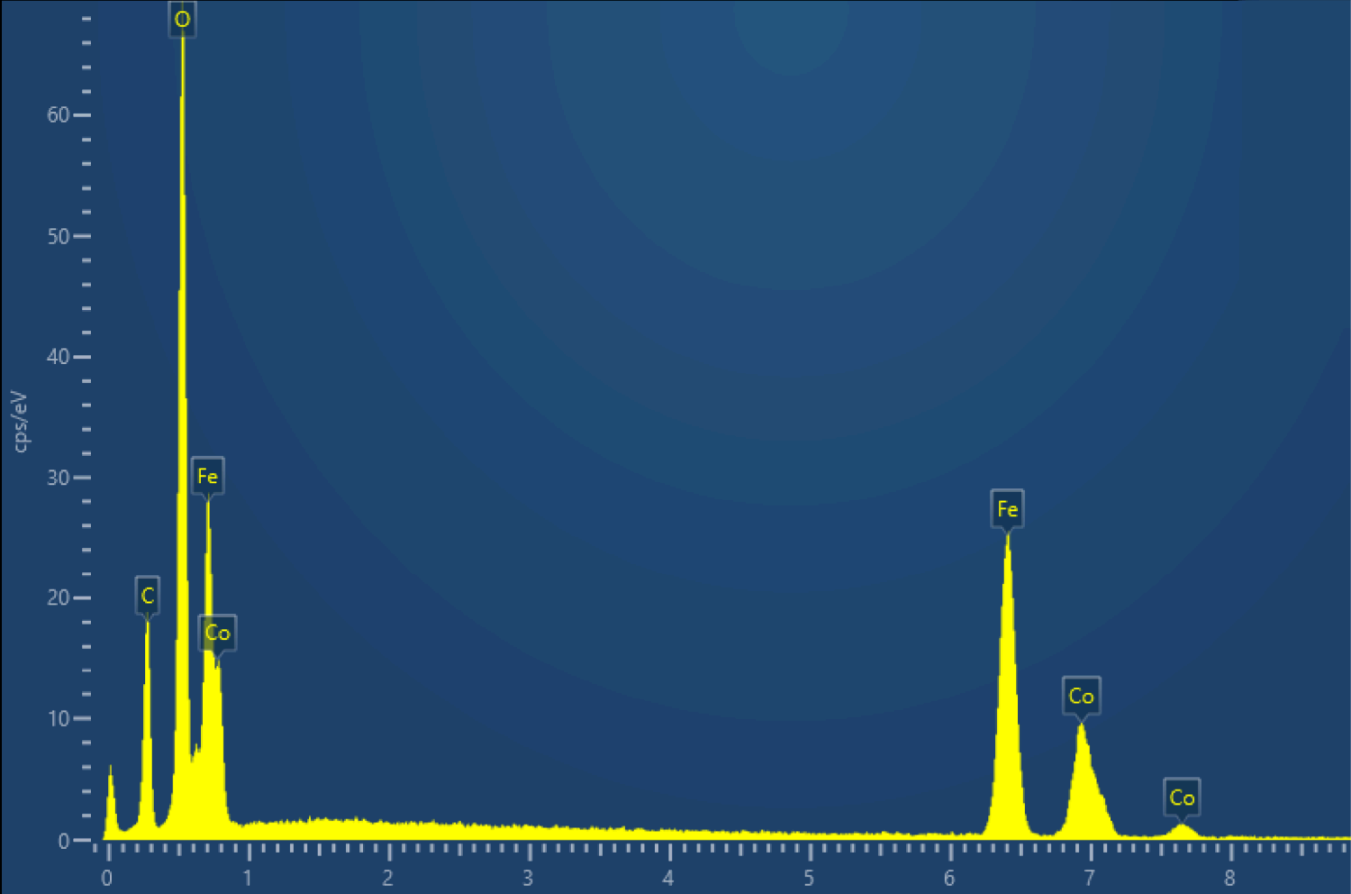
EDS data of cobalt ferrites.

The microscopic morphology of cobalt ferrite was visualized under FE-SEM. The microscopic morphology of cobalt ferrites prepared by the coprecipitation method (Figure 4) can be visualized from the original FE-SEM images showing irregular mixed spheres with hierarchical micro- and nanostructures. It includes a micrometer size morphology, irregular spherical shape, and slight agglomeration. These shapes are similar to the results shown by N. Hosni^52,53^ et al. Micro- and nanoparticles with hierarchical structures have been reported as another promising new strategy for the preparation of functional materials.^54^ The hierarchical structure can effectively expand the diversity of adsorbents and plays an important role in the adsorption of microplastics and organic solvents (Figure 4a,b), which can be used for oil–water separation. The average grain size obtained by FE-SEM graphical measurements was compared with that calculated by XRD. It was found that FE-SEM showed a larger grain size, which may be due to the agglomeration of the nanoparticles. It is attributed to the small size of the nanoparticles and enormous surface energy. It could also be due to the magnetic interactions between the nanoparticles.^55^ In addition, FE-SEM-EDS elemental mapping images show that the elements Fe, Co, and O are uniformly distributed throughout the area. Meanwhile, the EDS semiquantitative results (Figure 5) showed the atomic content ratio of Co:Fe was 1:2.4, which coincided with the analytical results of HR-XPS and proved the combination of CoFe_2_O_4_.

### Magnetic Properties

**Figure 6:**
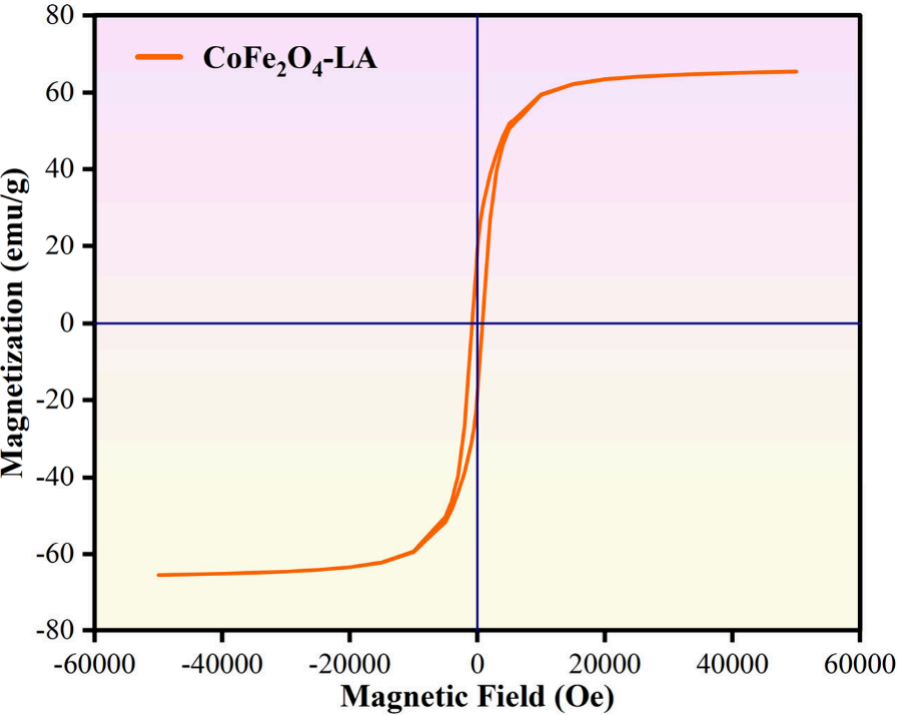
Magnetic hysteresis loops of surface functionalized cobalt ferrite.

Figure 6 shows the hysteresis loop of cobalt ferrite modified with lauric acid measured at 300 K under an external magnetic field of −60000 Oe ≤ *H* ≤ 60000 Oe. This study’s surface functionalized cobalt ferrite has a saturation magnetic field strength of 65.52 emu/g, a residual magnetization (Mr) of 18.79 emu/g, and a coercive force of 799.83 Oe. The coercive force (800 Oe) of cobalt ferrite prepared by other researchers using the coprecipitation method is similar.^56,57^ The saturation magnetization of cobalt ferrite is related to its crystallinity and particle size.^58^ The variation of coercive force with particle size can be explained in terms of crystal domain structure, critical diameter, strain, magnon crystal anisotropy, and crystal shape anisotropy.^59^ The hysteresis loop results indicate that surface functionalized cobalt ferrite has soft magnetic properties. The coercive force value is small and easy to magnetize and demagnetize, and the slight magnetic loss is helpful for high-frequency and repeated use of materials.^60^ Therefore, this cobalt ferrite can be easily separated from the aqueous solution by an external magnetic field to remove microplastics from water quickly, conveniently, and efficiently.^61^

### Wetting Properties

#### Contact Angle (CA) Measurements

**Figure 7:**
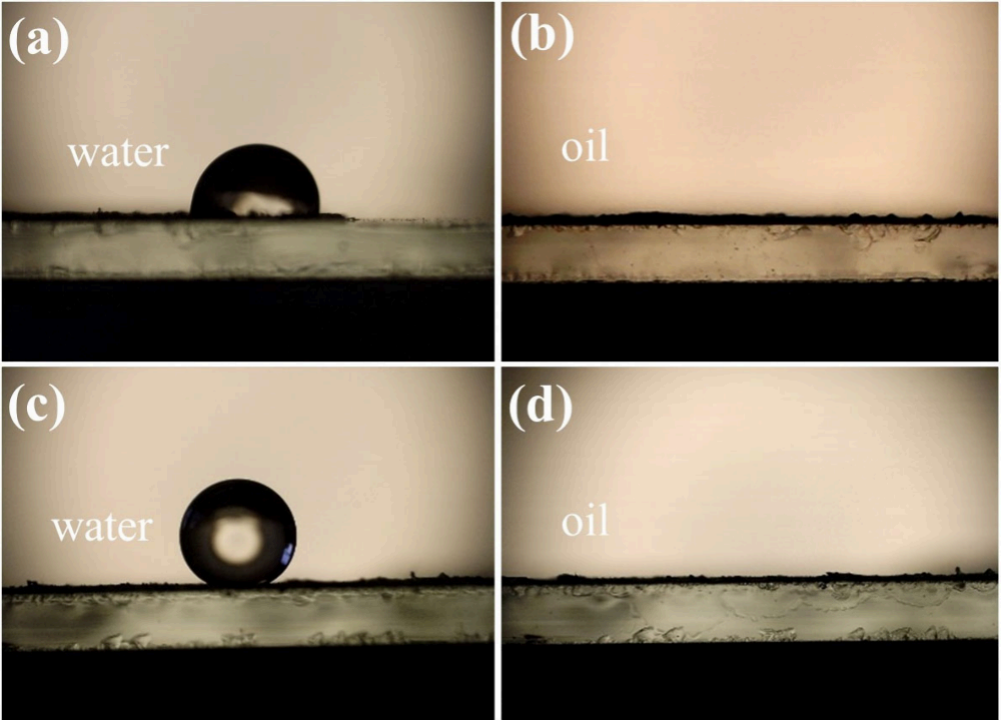
(a) WCA of powder before modification; (b) OCA of powder before modification; (c) WCA of powder after modification; (d) OCA of powder after modification.

Contact angle is a critical parameter in determining the surface superhydrophobicity. The WCA of cobalt ferrites was measured before and after the lauric acid surface modification process to determine the difference between the wetting states (Figure 7). The surface contact angle of the powder without lauric acid modification was 94.8°, exhibiting a hydrophobic surface. After modification with lauric acid, the contact angle of the powder increased to 157.3°, exhibiting a superhydrophobic surface. Based on XPS, the C–H group of lauric acid can effectively reduce the surface energy of cobalt ferrites.^37,44,62^Figure 7b and Figure 7d show that surface modification with lauric acid does not affect the oleophilicity of cobalt ferrites. The cobalt ferrites still exhibit superoleophilic properties. Contact angle measurements indicate that the cobalt ferrites were superhydrophobic and superoleophilic, which would be helpful in applications such as water–oil separation and microplastics removal.

**Figure 8:**
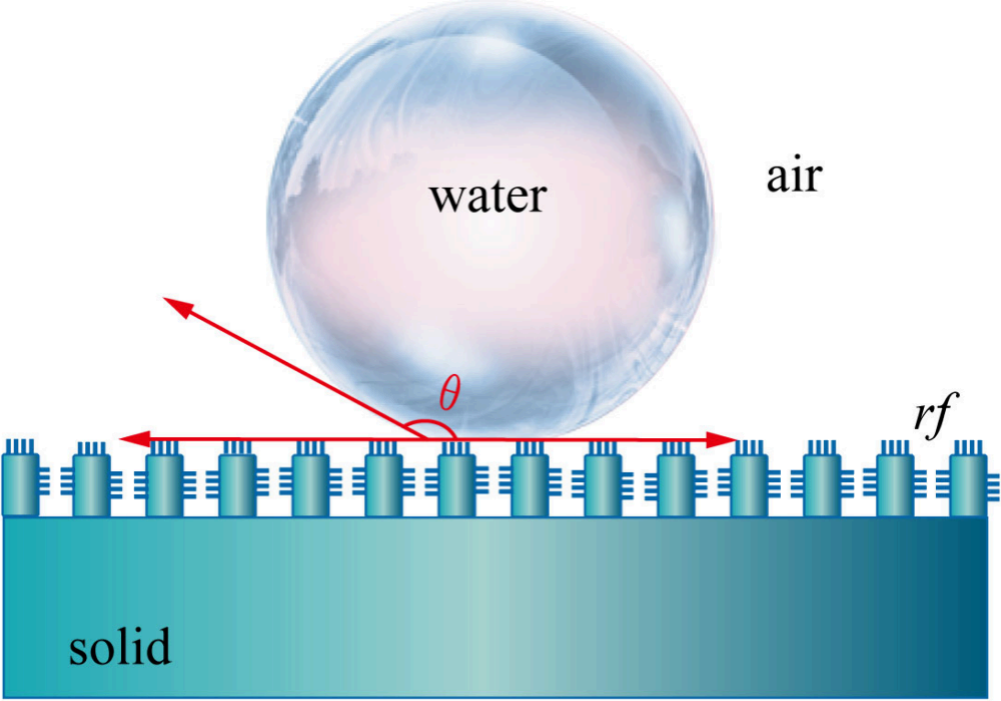
Cassie–Baxter state.

One of the challenges facing superhydrophobic surfaces is the gradual decrease in surface contact angle after a certain number of separation cycles.^63^ However, stabilized superhydrophobic properties are important for water–oil separation and hopefully for microplastics removal. It is worth mentioning that cobalt ferrites modified by lauric acid surface modification have stable superhydrophobic properties. The surface remains superhydrophobic even after six months or more of exposure to air. As shown in Figure 8, we can explain the water-repellent properties of cobalt ferrites with the help of the Cassie–Baxter state eq 7.^64,65^ For the combination of irregular spherical, nanolayered cobalt ferrite and surface lauric acid long-chain modification, when the cobalt ferrite comes in contact with water droplets, these droplets are in a solid–liquid–air nonwetting composite state in which the layered structure located in the cobalt ferrite traps tiny air pockets^66^
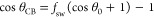
7where *f*_sw_ is the fraction of the projected area of the solid surface under the drop that is wet by the liquid.^67,68^*f*_sw_ of lauric-acid-modified cobalt ferrite was calculated to be 0.085, indicating that about 92% of the area was trapped with air.

#### Durability Test

**Figure 9:**
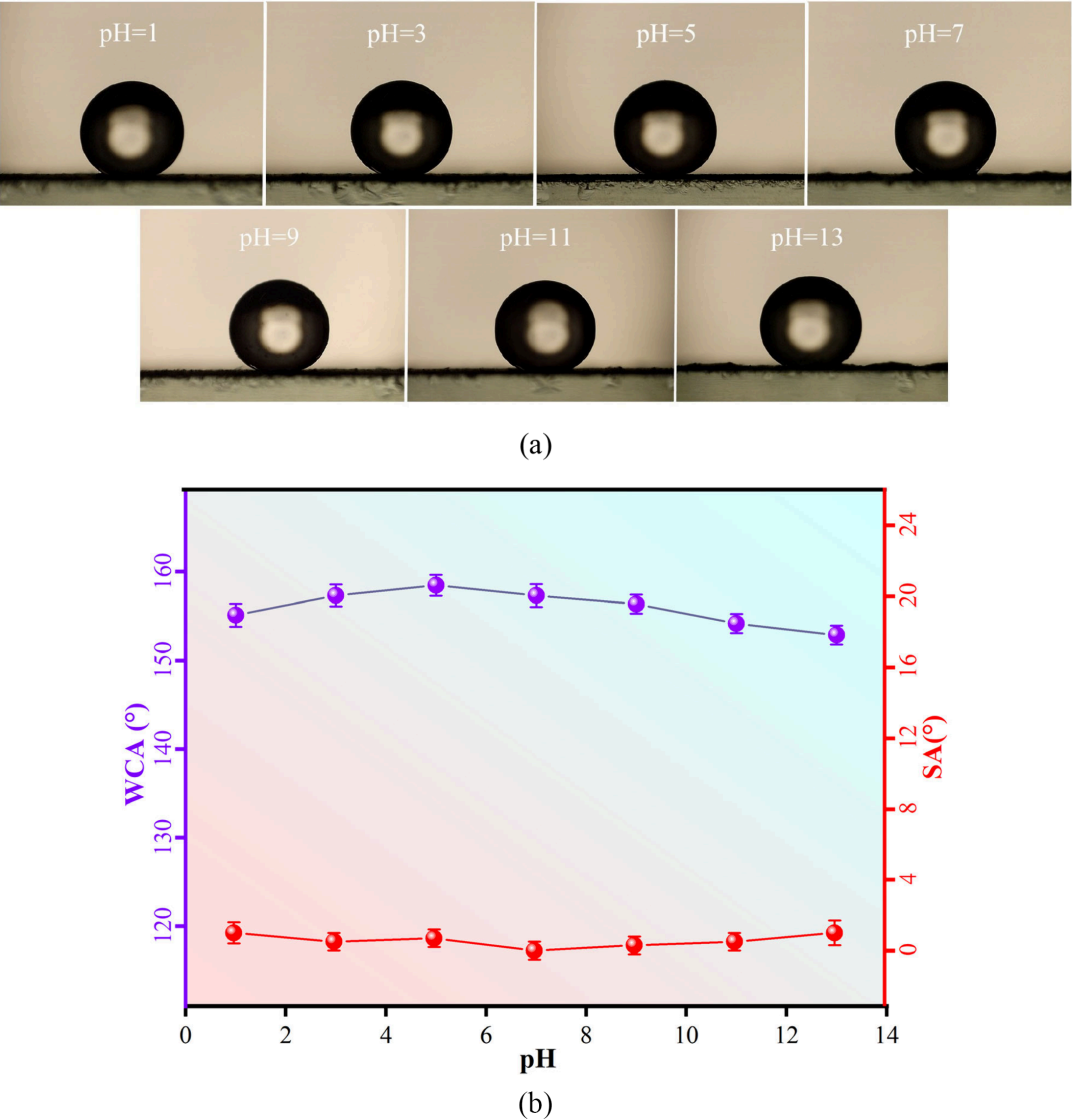
(a) Surface contact angle tested under different pH solutions. (b) Results of surface contact angle under different pH solutions. Error bars represent standard deviations, and statistical significance was assessed using one-way ANOVA (*p* < 0.05). The 95% confidence intervals are shown to illustrate the precision of the measurements.

Further, different pH solutions were prepared with HCl or NaOH to evaluate the wettability behavior of cobalt ferrites under different pH conditions. Figure 9 shows the effect of water droplets of different pH on the wettability of superhydrophobic cobalt ferrite powder. Overall, the surface contact angles of cobalt ferrites measured at water droplets of different pH are greater than 150° and have acceptable surface stability. The surface contact angle of cobalt ferrites tends to increase in the pH range of 1–3. The highest value is reached at pH equal to 5, which is 158.5°. In the pH range of 7–13, the surface contact angle decreased slightly, and was measured to be 152.8° at a pH of 13 in an aqueous solution. The SA value of cobalt ferrite tested in different pH solutions did not fluctuate significantly and was between 0 and 1°. The test results show that cobalt ferrites are superhydrophobic and well-tolerant to acidic and alkaline environments. This is mainly due to the synergistic effect of the microscopic layered structure of cobalt ferrite and the hydrophobic end of the surface modification. Therefore, when the liquid medium is in contact with cobalt ferrite, the microscopic rough surface can trap air to form an air cushion, which, together with the lauric acid modification to reduce its surface energy, greatly reduces the contact between cobalt ferrite and the liquid medium.

### Oil/Water Separation

**Figure 10:**
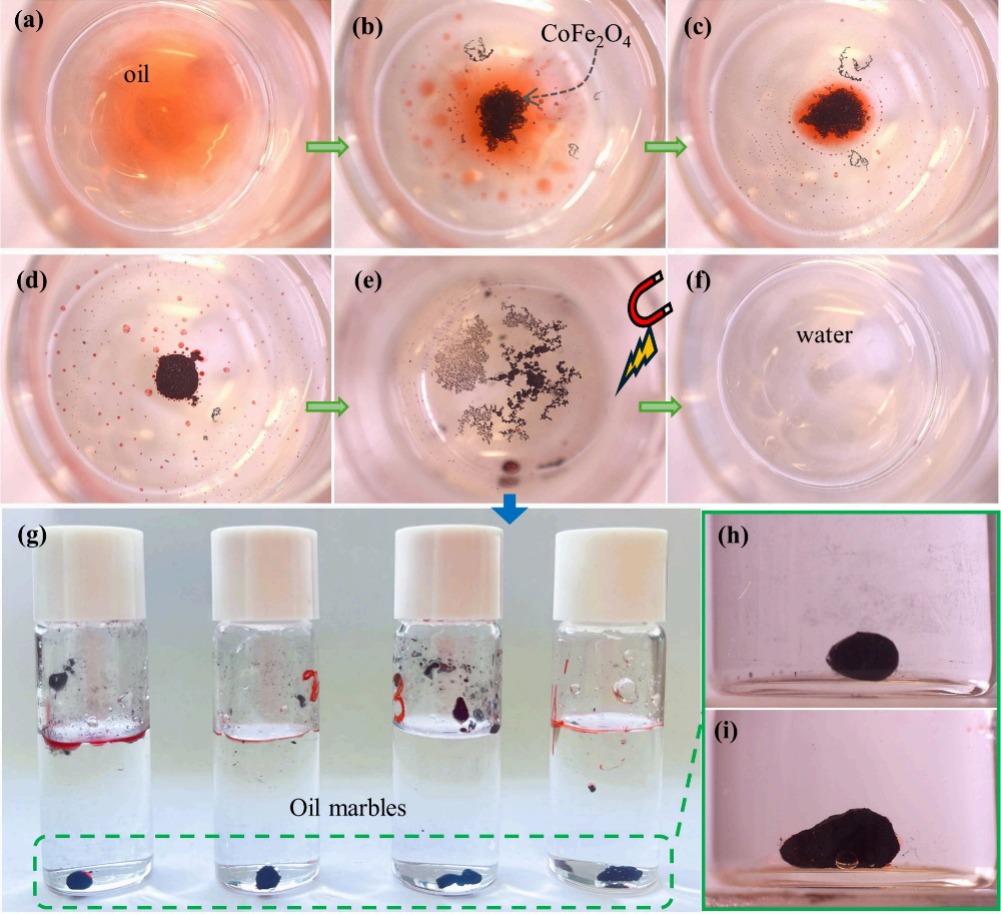
Process diagram of water–oil separation using cobalt ferrites: (a) dropping oil dyed with oil red into water, (b) adding cobalt ferrite, (c, d) the process of cobalt ferrite adsorbing oil, (e) using permanent magnets to adsorb cobalt ferrite, (f) water after removing the cobalt ferrite that adsorbs oil, (g–i) after cobalt ferrite adsorbs oil, oil marbles are formed in water.

The surface functionalized cobalt ferrites were known to have selective wettability (superoleophilic and superhydrophobic) by a surface contact angle test. To investigate the application potential and separation efficiency of the prepared cobalt ferrite for water–oil separation, four different organic solvents (as oils) were used for water–oil separation tests. They are hexane, petroleum ether, xylene, and olive oil. The separation process is shown in Figure 10, where 20 μL of oil red coloring droplets was first put into water. Then, a small amount (10–32 mg) of cobalt ferrite powder was added. The cobalt ferrite powders and the water–oil mixture were allowed to stand for 10 min. During this time, the area of the oil on the water surface was gradually decreased as the oil coalesced with the cobalt ferrite powder, until the red color on the water’s surface completely disappeared. Interestingly, after capturing many oil droplets, we observed that the cobalt ferrite forms spheres under water, termed “oil marbles”,^69−72^ which settle to the bottom of the glass bottle. Oil marbles are produced by cobalt ferrites, encapsulating oil particles. Finally, the cobalt ferrites were recovered by using a magnet. The cobalt ferrites were weighed and calculated.

**Figure 11:**
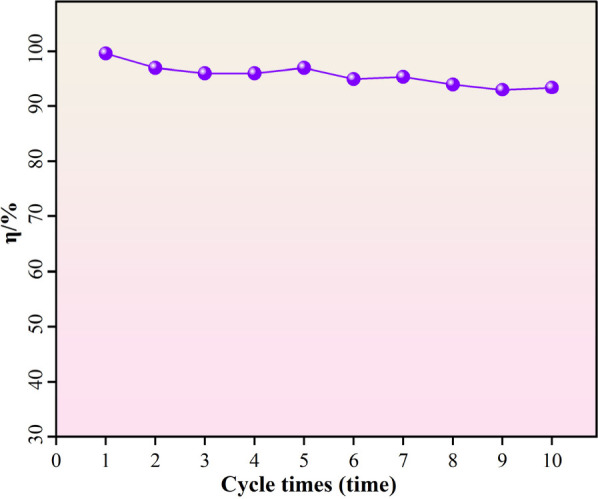
Separation efficiency of cobalt ferrites during the oil/water separation cycle.

According to eqs 2 and 3, the separation efficiency and adsorption capacity of cobalt ferrite powder for oil–water separation can be calculated, as presented in Table 1. As far as the separation efficiency is concerned, the efficiency of the powder in separating the four water–oil mixtures is higher than 94.2%. Among them, the separation of hexane and water mixtures was more excellent, with a separation efficiency of 99.6%. In terms of adsorption capacity, cobalt ferrite powder has excellent adsorption capacity for olive oil, which is 1.45 g/g. In addition, the accumulation of superhydrophobic particles in an aqueous solution significantly reduces the adsorption capacity, which is one of the main challenges in oil/water separation.^69^ Further, we take hexane as an example to study the separation efficiency of hexane for 10 times microplastic removal. The test results are listed in Figure 11. The efficiency of the 10 times test results was stable and kept above 93%. From the separation results, it can be judged that the powder has the ability to separate light oil, heavy oil, and water mixtures.

**Table 1:** Separation of Different Water–Oil Mixtures Performed by Cobalt Ferrite

	1-hexane	2-petroleum ether	3-xileno	4-olive oil
*Q* (g/g)	0.59	0.47	0.81	1.45
η (%)	99.6%	99.2%	96.0%	94.2%

Cobalt ferrites offer significant advantages in performance, efficiency, and cost compared to traditional filtration methods. While filtration technology can achieve high oil–water separation efficiency,^73^ membrane-based methods are prone to scaling and clogging, leading to frequent replacements and increased costs.^74−76^ In contrast, magnetic and superhydrophobic cobalt ferrites effectively absorb oil and microplastics without clogging. They maintain high separation efficiency over long periods and are reusable, reducing long-term costs. Thus, cobalt ferrites are a promising alternative for complex oil–water separation tasks.

### Microplastic Removal

**Figure 12:**
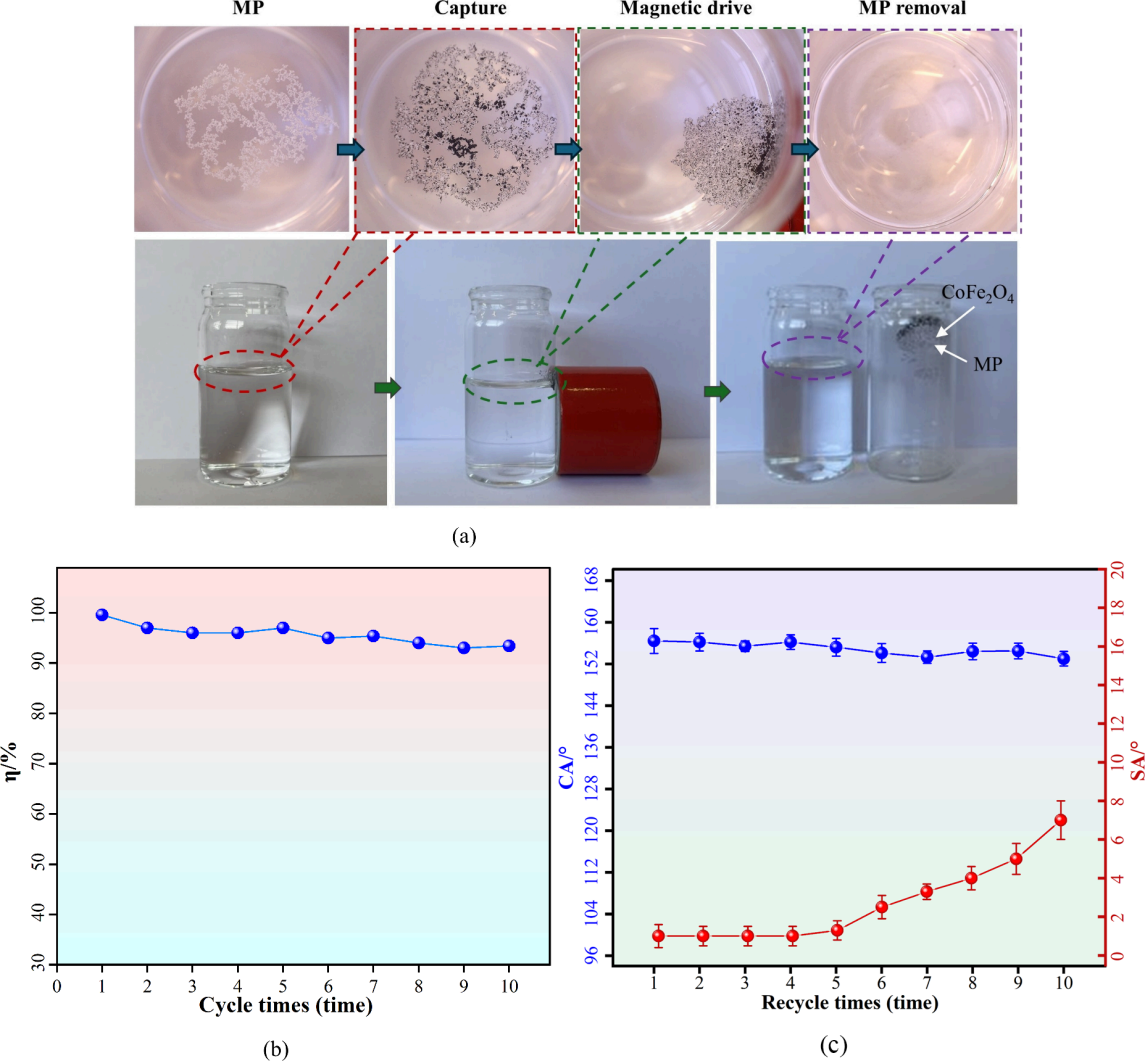
Overview of microplastic removal studies: (a) microplastic removal experimental process; (b) removal efficiency of microplastics by cobalt ferrites; (c) surface contact angle measurement results during the microplastic removal cycle. Error bars represent standard deviations, and statistical significance was assessed using one-way ANOVA (*p* < 0.05). The 95% confidence intervals are shown to illustrate the precision of the measurements.

As shown in Figure 12, we conducted experiments to remove different microplastic concentrations using cobalt ferrites with the help of permanent magnets. After each microplastic removal, the cobalt ferrites were washed with ethanol, dried in a fume hood, and recycled. The microplastic removal efficiency in each experiment was calculated, and the surface contact angle of the recycled cobalt ferrites was tested. As can be seen from the flowchart of the microplastic removal experiments (Figure 12a), after adding cobalt ferrites, microplastics formed flocs with cobalt ferrites.^77^ Then, the permanent magnet attracted the cobalt ferrites, and the microplastics approached the permanent magnet, together with the cobalt ferrites. The surface functionalization of lauric acid resulted in stable superhydrophobic properties on the surface of the cobalt ferrites. The cobalt ferrites modified by surface functionalization of lauric acid are subjected to spatial interference caused by long chain structures attached to the surface of cobalt ferrites at the moment of contact with water.^37^ The removal ability of cobalt ferrites at different concentrations of microplastics was tested. It can be seen from Figure 12b that the use of 0.01 g of cobalt ferrites efficiently adsorbs microplastics at concentrations ranging from 0.1 to 0.9 mmol/mL. The removal efficiency was up to 100%. Calculate the capture capacity and removal efficiency of the cobalt ferrites on microplastics according to eqs 4 and 5. The results are shown in Table 2. The removal efficiency was close to 100% at different microplastic concentrations. The ability of cobalt ferrites to capture microplastics increases with an increase in microplastic concentration. When there is 0.9 mmol/mL of microplastics in the aqueous solution and 0.01 g of cobalt ferrite is put into the solution, the capture capacity of microplastics is 2.56 g/g.

**Table 2:** Removal of Different Microplastic Concentrations by Cobalt Ferrite

MPs (mmol/mL)	0.1	0.3	0.5	0.7	0.9
Capture capacity (g/g)	1.31	1.44	1.54	2.36	2.56
ξ (%)	99%	99%	99%	99%	99%

The recycling of the pellets was carried out according to the methodology mentioned in the Experimental Section. In this regard, the contact angle of the particles was measured in different cycles, and in addition, the average microplastic removal efficiency in 10 microplastic cycling experiments was up to 98% as shown in Figure 12b. Even after 10 cycles of microplastic removal (Figure 12c), the surface contact angle of cobalt ferrites was still greater than 150°, presenting stable and excellent superhydrophobic properties. After the first five microplastic removal cycles, the surface roll angle of cobalt ferrite did not change significantly and was close to 0°. It was not until the sixth microplastic removal that the roll angle of cobalt ferrite increased to about 2.6°. After ten microplastic removal cycles, the surface roll angle of cobalt ferrite increased to about 7°. Although there was a small mass loss of cobalt ferrites after each recycling cycle, it was within acceptable limits. Due to the stability and superhydrophobicity of the modified particles at different pH values, there was no significant difference in the adsorption capacity of the particles in different cycles. Multiple recycling cycles did not affect the performance of the cobalt ferrite-captured microplastics. This is attributed to the functionalization of cobalt ferrite with lauric acid, which enables excellent and stable superhydrophobicity on its surface.^37^ It is essential to mention that going through multiple cycles of the recycling process does not affect the microplastic removal performance or superhydrophobic properties of cobalt ferrites in any way. The stability of cobalt ferrite modified by lauric acid functionalization may be related to the formation of hydrogen bonds between lauric acid and cobalt ferrite. This is attributed to the unique nanoflower-like layered structure of cobalt ferrite coupled with spatial interference induced by the long chains of functionalized modified lauric acid.

The above microplastic removal experimental results indicate that the functionalized cobalt ferrites have excellent microplastic removal efficiency, tenacious superhydrophobic properties, and recyclability. The recyclability property will increase the number of environmental applications of cobalt ferrites.

### Mechanism of Microplastic Removal

Fundamentally, the interaction between cobalt ferrites and microplastics floating on the water surface is caused by the wetting behavior at the interface between the solid phase, liquid phase, and gas phase.^78^ Rius-Ayra et al. used the DLVO theory to explain the mechanism of microplastic removal in water–oil systems and found that van der Waals forces play a key role in microplastic removal.^40,79^ Gu et al.^80^ used the DLVO theory to explore the adsorption phenomenon between *Thiobacillus ferrooxidans* and chalcopyrite. In our case, cobalt ferrite can be used in the water phase to directly remove microplastics. Microplastics and cobalt ferrite are hydrophobic substances, and the hydrophobic effect of water on both cannot be ignored. Therefore, in addition to considering van der Waals forces and double layer interactions, hydrophobic effects are also an important influencing factor. We use the extended DLVO theory to explain the mechanism of the interaction between cobalt ferrite and microplastics. The total interaction potential energy *V*_T_ between the two is equal to the sum of van der Waals forces-attraction *V*_W_, double layer interaction energy-repulsion *V*_E_, and hydrophobic effects-attraction *V*_H_.^81−83^
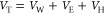
8

The van der Waals force between cobalt ferrite and microplastics is always mutually attractive.^84^ When the distance between particles is minimal, it promotes particle aggregation. According to research reports, the pH corresponding to the zero-zeta potential of cobalt ferrites is about 6.5.^26,85,86^ The pH of tap water used in microplastic removal testing is about 7.84 ± 0.08. Both nano- and microsized plastics carry negative charges.^77,87^ According to the principle of electrostatic interaction, there is an electrostatic repulsion between two substances with the same charge. Interestingly, cobalt ferrite with negative charges on the surface can attract and capture microplastics that also carry negative charges. In the Contact Angle (CA) Measurements section, we tested the surface contact angle of cobalt ferrite in aqueous solutions of different pH values and found that the superhydrophobic properties of cobalt ferrite are adapted to aqueous solutions of different acid and base types. Hydrophobic interactions can also be understood as the minimization of the interfacial energy of the water system. In general, hydrophobic interactions can be explained by the tendency of nonpolar molecular surfaces to aggregate in aqueous solutions (or to be expelled from water into aggregates). Water plays an important role in the hydrophobic interaction due to discrete nanoscale surface heterogeneity and hydrophobic/solvation effects (hydrophobic forces), which arise from the structural competition between hydrogen bonds in bulk water and hydrogen bonds in interfacial water.^88^ The interfacial tension between water and nonpolar materials is typically around 51 mJ/m^2^.

**Figure 13:**
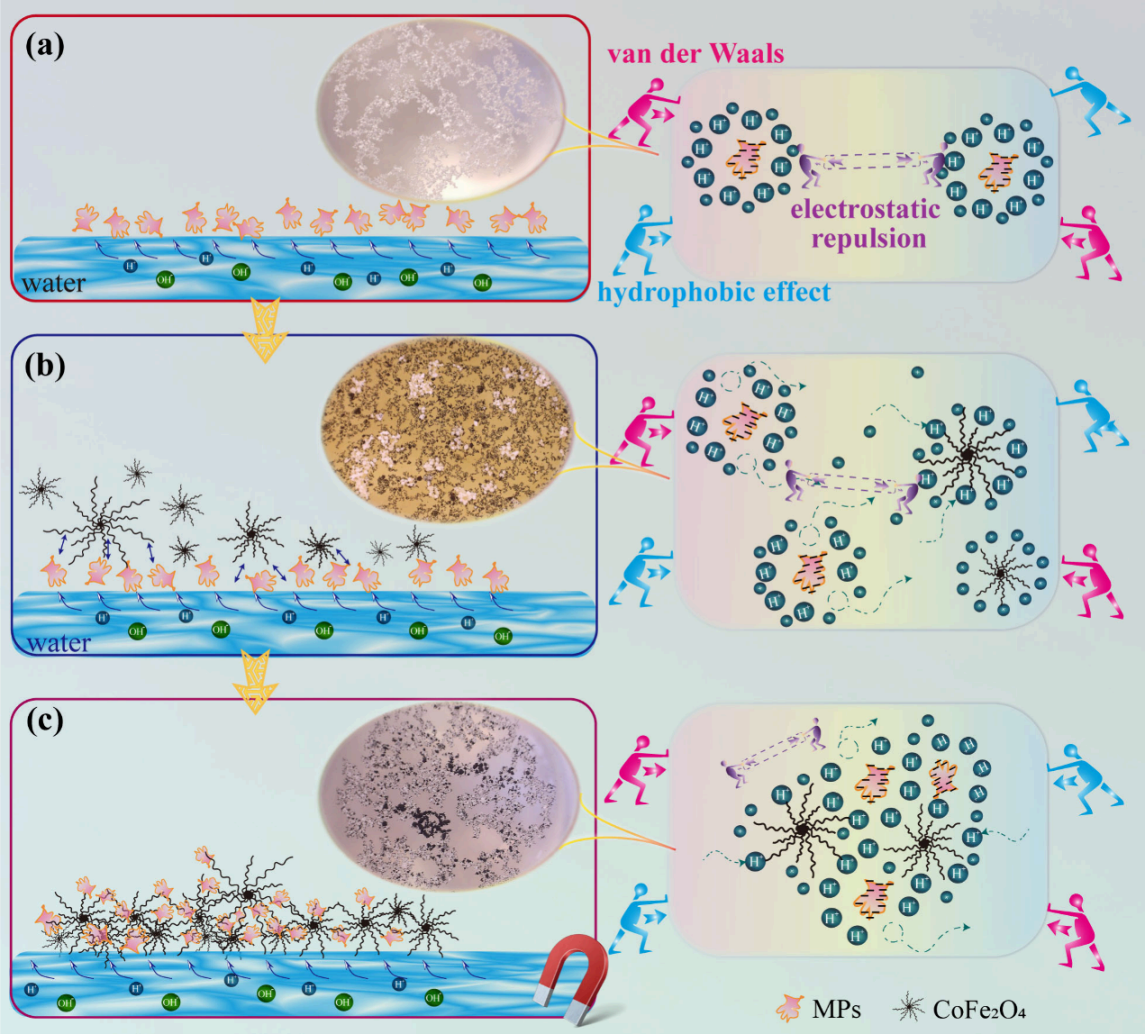
Mechanism of microplastics removal by cobalt ferrites: (a) microplastics only; (b) addition of cobalt ferrite to capture microplastics; (c) cobalt ferrite capturing microplastics.

Microplastics can be considered as electrostatically stable and highly dispersed colloids.^89,90^ The water surface is usually charged by ionization or dissociation of surface groups. The H^+^ released from water and the negative charge on the surface of microplastics form a stable double layer. For bare microplastics, the van der Waals attraction and hydrophobic force are greater than the electrostatic repulsion, resulting in a net interparticle attraction and negative interaction energy. When only microplastics exist on the water surface (Figure 13a), the microplastics are in a stable state at the water–air–solute interface under the synergistic effect of hydrophobic interaction and electrostatic repulsion between microplastics.^91^

When magnetic cobalt ferrite is added (Figure 13b), the surface area of hydrophobic solutes in water increases, the water–solute interface tends to be stable, the hydrogen bonds in water are broken, and exclusivity appears in order to minimize the interface energy. Microplastics will swim toward cobalt ferrite. This is because the second layer of hydrated positive ions free on the surface of microplastics is not stable. Attracted by the negatively charged cobalt ferrite, the hydrated positive ions approach it to form a double electric layer structure. In this process, the double electric layer structure composed of some microplastics and hydrated positive ions is destroyed. Cobalt ferrite has a large specific surface area due to its nanoscale size. Therefore, compared with microplastics, the number of hydrated positive ions bound to the surface of cobalt ferrite is greater. Therefore, under the attraction of hydrated positive ions, microplastics approach cobalt ferrite to share hydrated positive ions and achieve a stable double electric layer structure. At this time, there is no simple electrostatic repulsion between microplastics and cobalt ferrite, but also electrostatic attraction due to shared hydrated positive ions. The interaction energy barrier between microplastics and cobalt ferrite is obviously attributed to electrostatic repulsion. The van der Waals force and hydrophobic attraction between microplastics and cobalt ferrite overcome the electrostatic repulsion, resulting in a net attraction and interaction energy (Figure 13c). As the concentration of hydrophobic substances (cobalt ferrite or microplastics) in water increases, the hydrophobic effect becomes dominant, driving the microplastic particles closer to the cobalt ferrite powder. As the two continue to move closer and the distance between them shrinks to 0.157 nm (critical distance),^92^ the van der Waals force gradually plays a key role, causing the microplastics to be closely adsorbed on the cobalt ferrite.

Therefore, the mechanism of microplastic capture can be explained as follows: cobalt ferrite, under the synergistic effect of van der Waals force and hydrophobic interaction, overcomes the barrier of electrostatic repulsion and captures microplastics floating in the water. The interfacial adsorption of microplastics and cobalt ferrites can reduce their contact area with water, decreasing the interfacial binding energy. Due to the long carbon chain modification and nanometer size on the surface, cobalt ferrites show excellent adsorption capacity for microplastics.^93^ Furthermore, under the attraction of a permanent magnet, the collection of microplastics and recovery of cobalt ferrite can be easily achieved.

## Conclusions

Environmental pollution caused by microplastics and its effects on human health have become a global concern. Recyclable superhydrophobic magnetic particles with micro- and nanostructures have been recognized as safe, practical, and easy-to-use potential tools for microplastic removal from the environment. We fabricated superhydrophobic CoFe_2_O_4_ particles using a facile coprecipitation method with lauric acid surface modification. Surface functionalized cobalt ferrite presented irregular mixed spheres with layered micro-nanostructures. XRD showed that the average diameter of cobalt ferrites was 62.5 nm. The saturation magnetic field strength of cobalt ferrites was 65.52 emu/g with low coercivity and soft magnetism. Cobalt ferrite exhibited superhydrophobic properties under different pH solution tests, revealing WCA = 157.3° and OCA = 0° for effective microplastic removal and oil–water separation. The superhydrophobic properties were also maintained after exposure to different pH solutions; WCA remained higher than 150° and SA values of cobalt ferrite were still between 0 and 1°. We also examined the suitability of superhydrophobic cobalt ferrites for separating various oils, including hexane, petroleum ether, xylene, and olive oil, from the oil–water mixtures. The oil–water separation experiments demonstrated the cobalt ferrites’ high separation efficiency and adsorption capacity: the separation efficiency of four oil/water mixtures was more than 94.2%. After 10 times oil(hexane)/water, the separation efficiency remained more than 93%. In addition, the cobalt ferrites maintained excellent stability in successive microplastic removal cycles. After ten cycles of plastic removal, the separation efficiency reached 99%. The absorption capacity reached 2.56 g/g. We proposed a possible mechanism for microplastic removal by cobalt ferrites based on EDLVO and interfacial energy minimization theory. Herein, we show that superhydrophobic CoFe_2_O_4_ particles are a promising environmental approach to remove oils and microplastics from water.

## References

[ref1] Thompson R. C. Microplastics in the Marine Environment: Sources, Consequences and Solutions. In Marine Anthropogenic Litter; Bergmann M., Gutow L., Klages M., Eds.; Springer International Publishing: Cham, Switzerland, 2015; pp 185–200.

[ref2] Talang R. P. N.; Polruang S.; Sirivithayapakorn S. Influencing factors of microplastic generation and microplastic contamination in urban freshwater. Heliyon 2024, 10 (9), e30021 10.1016/j.heliyon.2024.e30021. 38707367 PMC11068644

[ref3] Chen J.; Wu J.; Sherrell P. C.; Chen J.; Wang H.; Zhang W.-x.; Yang J. How to Build a Microplastics-Free Environment: Strategies for Microplastics Degradation and Plastics Recycling. Advanced Science 2022, 9 (6), 2103764–2103799. 10.1002/advs.202103764. 34989178 PMC8867153

[ref4] Sarbatly R.; Krishnaiah D.; Kamin Z. A review of polymer nanofibres by electrospinning and their application in oil-water separation for cleaning up marine oil spills. Mar. Pollut. Bull. 2016, 106 (1), 8–16. 10.1016/j.marpolbul.2016.03.037. 27016959

[ref5] Bhardwaj L. K.; Rath P.; Yadav P.; Gupta U. Microplastic contamination, an emerging threat to the freshwater environment: a systematic review. Environmental Systems Research 2024, 13, 8 10.1186/s40068-024-00338-7.

[ref6] Chen J.; Wu J.; Sherrell P. C.; Chen J.; Wang H.; Zhang W.; Yang J. How to Build a Microplastics-Free Environment: Strategies for Microplastics Degradation and Plastics Recycling. Advanced Science 2022, 9 (6), 2103764 10.1002/advs.202103764. 34989178 PMC8867153

[ref7] Tirkey A.; Pandey M.; Tiwari A.; Sahu R. L.; Kukkar D.; Dubey R.; Kim K.-H.; Pandey S. K. Global distribution of microplastic contaminants in aquatic environments and their remediation strategies. Water Environment Research 2022, 94 (12), e10819 10.1002/wer.10819. 36539344

[ref8] Sun J.; Dai X.; Wang Q.; van Loosdrecht M. C. M.; Ni B. Microplastics in wastewater treatment plants: Detection, occurrence and removal. Water Res. 2019, 152, 21–37. 10.1016/j.watres.2018.12.050. 30660095

[ref9] Li L.; Xu G.; Yu H.; Xing J. Dynamic membrane for micro-particle removal in wastewater treatment: Performance and influencing factors. Science of The Total Environment 2018, 627, 332–340. 10.1016/j.scitotenv.2018.01.239. 29426156

[ref10] Akbal F.; Camcı S. Copper, chromium and nickel removal from metal plating wastewater by electrocoagulation. Desalination 2011, 269 (1), 214–222. 10.1016/j.desal.2010.11.001.

[ref11] Shirasaki N.; Matsushita T.; Matsui Y.; Marubayashi T. Effect of aluminum hydrolyte species on human enterovirus removal from water during the coagulation process. Chemical Engineering Journal 2016, 284, 786–793. 10.1016/j.cej.2015.09.045.

[ref12] Urbanek A. K.; Rymowicz W.; Mironczuk A. M. Degradation of plastics and plastic-degrading bacteria in cold marine habitats. Appl. Microbiol. Biotechnol. 2018, 102, 7669–7678. 10.1007/s00253-018-9195-y. 29992436 PMC6132502

[ref13] Mayorga-Burrezo P.; Mayorga-Martinez C. C.; Pumera M. Photocatalysis dramatically influences motion of magnetic microrobots: Application to removal of microplastics and dyes. J. Colloid Interface Sci. 2023, 643, 447–454. 10.1016/j.jcis.2023.04.019. 37086534

[ref14] Zulfiqar U.; Hussain S. Z.; Subhani T.; Hussain I.; Habib-ur-Rehman Mechanically robust superhydrophobic coating from sawdust particles and carbon soot for oil/water separation. Colloids Surf., A 2018, 539 (20), 391–398. 10.1016/j.colsurfa.2017.12.047.

[ref15] Misra A.; Zambrzycki C.; Kloker G.; Kotyrba A.; Anjass M. H.; Franco Castillo I.; Mitchell S. G.; Guttel R.; Streb C. Water Purification and Microplastics Removal Using Magnetic Polyoxometalate-Supported Ionic Liquid Phases (magPOM-SILPs). Angew. Chem., Int. Ed. 2020, 59 (4), 1601–1605. 10.1002/anie.201912111. PMC7004052 31639241

[ref16] Herrmann S.; De Matteis L.; de la Fuente J. M.; Mitchell S. G.; Streb C. Removal of Multiple Contaminants from Water by Polyoxometalate Supported Ionic Liquid Phases (POM-SILPs). Angew. Chem., Int. Ed. 2017, 56 (6), 1667–1670. 10.1002/anie.201611072. 28079959

[ref17] Guo S.; Jiao P.; Dan Z.; Duan N.; Zhang J.; Chen G.; Gao W. Synthesis of magnetic bioadsorbent for adsorption of Zn(II), Cd(II) and Pb(II) ions from aqueous solution. Chem. Eng. Res. Des. 2017, 126, 217–231. 10.1016/j.cherd.2017.08.025.

[ref18] Rius-Ayra O.; Biserova-Tahchieva A.; López-Jiménez I.; Llorca-Isern N. Superhydrophobic and nanostructured CuFeCo powder alloy for the capture of microplastics. Colloids Surf., A 2021, 627, 127075 10.1016/j.colsurfa.2021.127075.

[ref19] Houshiar M.; Zebhi F.; Razi Z. J.; Alidoust A.; Askari Z. Synthesis of cobalt ferrite (CoFe2O4) nanoparticles using combustion, coprecipitation, and precipitation methods: A comparison study of size, structural, and magnetic properties. J. Magn. Magn. Mater. 2014, 371, 43–48. 10.1016/j.jmmm.2014.06.059.

[ref20] Hajalilou A.; Mazlan S. A.; Abbasi M.; Lavvafi H. Fabrication of spherical CoFe2O4 nanoparticles via sol-gel and hydrothermal methods and investigation of their magnetorheological characteristics. RSC Adv. 2016, 6 (92), 89510–89522. 10.1039/C6RA13493A.

[ref21] Cabañas A.; Poliakoff M. The continuous hydrothermal synthesis of nano-particulate ferrites in near critical and supercritical water. J. Mater. Chem. 2001, 11 (5), 1408–1416. 10.1039/b009428p.

[ref22] Lee J.-G.; Park J. Y.; Kim C. S. Growth of ultra-fine cobalt ferrite particles by a sol-gel method and their magnetic properties. Journal of Materials Science 1998, 33, 3965–3968. 10.1023/A:1004696729673.

[ref23] Sun S.; Zeng H.; Robinson D. B.; Raoux S.; Rice P. M.; Wang S. X.; Li G. Monodisperse MFe2O4 (M = Fe, Co, Mn) Nanoparticles. J. Am. Chem. Soc. 2004, 126 (1), 273–279. 10.1021/ja0380852. 14709092

[ref24] Bensebaa F.; Zavaliche F.; L’Ecuyer P.; Cochrane R. W.; Veres T. Microwave synthesis and characterization of Co-ferrite nanoparticles. J. Colloid Interface Sci. 2004, 277 (1), 104–110. 10.1016/j.jcis.2004.04.016. 15276045

[ref25] Manova E.; Kunev B.; Paneva D.; Mitov I.; Petrov L.; Estournes C.; Rehspringer J.-L.; Kurmoo M. Mechano-Synthesis, Characterization, and Magnetic Properties of Nanoparticles of Cobalt Ferrite, CoFe2O4. Chem. Mater. 2004, 16 (26), 5689–5696. 10.1021/cm049189u.

[ref26] de Vicente J.; Delgado A. V.; Plaza R. C.; Duran J. D. G.; Gonzalez-Caballero F. Stability of Cobalt Ferrite Colloidal Particles. Effect of pH and Applied Magnetic Fields. Langmuir 2000, 16 (21), 7954–7961. 10.1021/la0003490.

[ref27] Blaskov V.; Petkov V.; Rusanov V.; Martinez Ll. M.; Martinez B.; Muñoz J. S.; Mikhov M. Magnetic properties of nanophase CoFe2O4 particles. J. Magn. Magn. Mater. 1996, 162 (2–3), 331–337. 10.1016/S0304-8853(96)00277-6.

[ref28] Lv C.; Wang H.; Liu Z.; Zhang W.; Wang C.; Tao R.; Li M.; Zhu Y. A sturdy self-cleaning and anti-corrosion superhydrophobic coating assembled by amino silicon oil modifying potassium titanate whisker-silica particles. Appl. Surf. Sci. 2018, 435, 903–913. 10.1016/j.apsusc.2017.11.205.

[ref29] Zhu S.; Deng W.; Su Y. Recent advances in preparation of metallic superhydrophobic surface by chemical etching and its applications. Chinese Journal of Chemical Engineering 2023, 61, 221–236. 10.1016/j.cjche.2023.02.018.

[ref30] Kim B.; Lee J.; Lee E.; Jeong K.; Seo J.-H. One-pot coatable fluorinated polyurethane resin solution for robust superhydrophobic anti-fouling surface. Prog. Org. Coat. 2024, 187, 108097 10.1016/j.porgcoat.2023.108097.

[ref31] Feng X. J.; Shi Y. L.; Yin X. L.; Wang X. Transparent superhydrophobic film with anti-fouling and anti-scaling ability by facile method of dip-coating SiO2 Sol. Materials Research Express 2022, 9 (1), 016404 10.1088/2053-1591/ac4aa4.

[ref32] Drelich J.; Chibowski E. Superhydrophilic and Superwetting Surfaces: Definition and Mechanisms of Control. Langmuir 2010, 26 (24), 18621–18623. 10.1021/la1039893. 21090661

[ref33] Law K.-Y. Definitions for Hydrophilicity, Hydrophobicity, and Superhydrophobicity: Getting the Basics Right. J. Phys. Chem. Lett. 2014, 5 (4), 686–688. 10.1021/jz402762h. 26270837

[ref34] Rius-Ayra O.; Biserova-Tahchieva A.; Llorca-Isern N. Removal of dyes, oils, alcohols, heavy metals and microplastics from water with superhydrophobic materials. Chemosphere 2023, 311, 137148 10.1016/j.chemosphere.2022.137148. 36351466

[ref35] Parvate S.; Dixit P.; Chattopadhyay S. Superhydrophobic Surfaces: Insights from Theory and Experiment. JOURNAL OF PHYSICAL CHEMISTRY B 2020, 124 (8), 1323–1360. 10.1021/acs.jpcb.9b08567. 31931574

[ref36] Wang Z.; Li Q.; She Z.; Chen F.; Li L. Low-cost and large-scale fabrication method for an environmentally-friendly superhydrophobic coating on magnesium alloy. J. Mater. Chem. 2012, 22 (9), 4097–4105. 10.1039/c2jm14475a.

[ref37] Huang F.; Li Q.; Ji G.; Tu J.; Ding N.; Qu Q.; Liu G. Oil/water separation using a lauric acid-modified, superhydrophobic cellulose composite membrane. Mater. Chem. Phys. 2021, 266, 124493 10.1016/j.matchemphys.2021.124493.

[ref38] Scherrer P. Bestimmung der Größe und der inneren Struktur von Kolloidteilchen mittels Röntgenstrahlen. Mathematisch-Physikalische Klasse 1918, 1918, 98–100.

[ref39] Rius-Ayra O.; Bouhnouf-Riahi O.; LLorca-Isern N. Superhydrophobic and Sustainable Nanostructured Powdered Iron for the Efficient Separation of Oil-in-Water Emulsions and the Capture of Microplastics. ACS Appl. Mater. Interfaces 2020, 12 (40), 45629–45640. 10.1021/acsami.0c13876. 32926613

[ref40] Rius-Ayra O.; Biserova-Tahchieva A.; Sansa-López V.; Llorca-Isern N. Superhydrophobic PDMS coated 304 stainless-steel mesh for the removal of HDPE microplastics. Prog. Org. Coat. 2022, 170, 107009 10.1016/j.porgcoat.2022.107009. PMC9097532 35465677

[ref41] Sebatini A. M.; Jain M.; Radha P.; Kiruthika S.; Tamilarasan K. Immobilized lipase catalyzing glucose stearate synthesis and their surfactant properties analysis. 3 Biotech 2016, 6, 1–8. 10.1007/s13205-016-0501-z. PMC5003783 28330256

[ref42] Huang R.; Wang L.; Lin Y.; Dong Y.; You D. Surface modification of carbonyl iron powders with silicone polymers in supercritical fluid to get higher dispersibility and higher thermal stability. Surf. Interface Anal. 2017, 49 (2), 79–84. 10.1002/sia.6061.

[ref43] Kong X.; Fang Z.; Bao X.; Wang Z.; Mao S.; Wang Y. Efficient hydrogenation of stearic acid over carbon coated Nisingle bondFe catalyst. J. Catal. 2018, 367, 139–149. 10.1016/j.jcat.2018.08.022.

[ref44] Rabbani Y.; Shariaty-Niassar M.; Ebrahimi S. A. S. The effect of superhydrophobicity of prickly shape carbonyl iron particles on the oil-water adsorption. Ceram. Int. 2021, 47 (20), 28400–28410. 10.1016/j.ceramint.2021.06.257.

[ref45] Gu Z.; Xiang X.; Fan G.; Li F. Facile Synthesis and Characterization of Cobalt Ferrite Nanocrystals via a Simple Reduction-Oxidation Route. J. Phys. Chem. C 2008, 112 (47), 18459–18466. 10.1021/jp806682q.

[ref46] He J.; Fu X.; Ni F.; Yang G.; Deng S.; Chen J. P.; Shen F. Quantitative assessment of interactions of hydrophilic organic contaminants with microplastics in natural water environment. Water Res. 2022, 224, 119024 10.1016/j.watres.2022.119024. 36099764

[ref47] Hu P.; Ren J.; Ren W.; Sun Y.; Yang H. The feasibility and mechanism of poly-aluminum/titanium silicate composite coagulants for the efficient removal of nano- and micro-sized plastics. Chemical Engineering Journal 2024, 482, 149095 10.1016/j.cej.2024.149095.

[ref48] Santhosh C.; Daneshvar E.; Kollu P.; Peraniemi S.; Grace A. N.; Bhatnagar A. Magnetic SiO2@CoFe2O4 nanoparticles decorated on graphene oxide as efficient adsorbents for the removal of anionic pollutants from water. Chemical Engineering Journal 2017, 322 (15), 472–487. 10.1016/j.cej.2017.03.144.

[ref49] Biesinger M. C.; Payne B. P.; Grosvenor A. P.; Lau L. W.M.; Gerson A. R.; Smart R. S. C. Resolving surface chemical states in XPS analysis of first row transition metals, oxides and hydroxides: Cr, Mn, Fe, Co and Ni. Appl. Surf. Sci. 2011, 257 (7), 2717–2730. 10.1016/j.apsusc.2010.10.051.

[ref50] Gu Z.; Xiang X.; Fan G.; Li F. Facile Synthesis and Characterization of Cobalt Ferrite Nanocrystals via a Simple Reduction-Oxidation Route. J. Phys. Chem. C 2008, 112 (47), 18459–18466. 10.1021/jp806682q.

[ref51] Briggs D.; Seah M. P. Practical Surface Analysis, Auger and X-ray Photoelectron Spectroscopy; Wiley: 1990; Vol. 2024, p 674.

[ref52] Hosni N.; Zehani K.; Bartoli T.; Bessais L.; Maghraoui-Meherzi H. Semi-hard magnetic properties of nanoparticles of cobalt ferrite synthesized by the co-precipitation process. J. Alloys Compd. 2017, 694, 1295–1301. 10.1016/j.jallcom.2016.09.252.

[ref53] Saremi A.; Mirkazemi S. M.; Sazvar A.; Rezaie H. Controlling magnetic and surface properties of cobalt ferrite nanoparticles: A comparison of co-precipitation and solvothermal synthesis methods. Solid State Sci. 2024, 148, 107432–107441. 10.1016/j.solidstatesciences.2023.107432.

[ref54] Rezk M.; Allam N. K. Impact of Nanotechnology on Enhanced Oil Recovery: A Mini-Review. Ind. Eng. Chem. Res. 2019, 58 (36), 16287–16295. 10.1021/acs.iecr.9b03693.

[ref55] Tei C.; Ohtaka M.; Kuwahara D. Elucidation of solid particle interfacial Phenomena in liquid sodium: Magnetic interactions on liquid metal and solid atoms at the solid interface. Chem. Phys. Lett. 2023, 829, 140755–140760. 10.1016/j.cplett.2023.140755.

[ref56] Gyergyek S.; Makovec D.; Kodre A.; et al. Influence of synthesis method on structural and magnetic properties of cobalt ferrite nanoparticles. J. Nanopart. Res. 2010, 12, 1263–1273. 10.1007/s11051-009-9833-5.

[ref57] Houshiar M.; Zebhi F.; Razi Z. J.; Alidoust A.; Askari Z. Synthesis of cobalt ferrite (CoFe2O4) nanoparticles using combustion, coprecipitation, and precipitation methods: A comparison study of size, structural, and magnetic properties. J. Magn. Magn. Mater. 2014, 371, 43–48. 10.1016/j.jmmm.2014.06.059.

[ref58] de Vicente J.; Delgado A. V.; Plaza R. C.; Duran J. D. G.; Gonzalez-Caballero F. Stability of Cobalt Ferrite Colloidal Particles. Effect of pH and Applied Magnetic Fields. Langmuir 2000, 16 (21), 7954–7961. 10.1021/la0003490.

[ref59] Nawale A. B.; Kanhe N. S.; Patil K.R.; Bhoraskar S.V.; Mathe V.L.; Das A.K. Magnetic properties of thermal plasma synthesized nanocrystalline nickel ferrite (NiFe2O4). J. Alloys Compd. 2011, 509 (12), 4404–4413. 10.1016/j.jallcom.2011.01.057.

[ref60] Darwish M.A.; Saafan S.A.; El- Kony D.; Salahuddin N.A. Preparation and investigation of dc conductivity and relative permeability of epoxy/Li-Ni-Zn ferrite composites. J. Magn. Magn. Mater. 2015, 385 (1), 99–106. 10.1016/j.jmmm.2015.02.068.

[ref61] Chella S.; Kollu P.; Komarala E. V. P R; Doshi S.; Saranya M.; Felix S.; Ramachandran R.; Saravanan P.; Koneru V. L.; Venugopal V.; Jeong S. K.; Nirmala Grace A. Solvothermal synthesis of MnFe2O4-graphene composite—Investigation of its adsorption and antimicrobial properties. Appl. Surf. Sci. 2015, 327 (1), 27–36. 10.1016/j.apsusc.2014.11.096.

[ref62] Wu Q.; Zhang W.; Qu C.; Yuan F.; Huang Y.; Chen X.; Zhang R. Lauric acid surface modified Co2+-ZnO@SSM composite membrane: Photocatalytic degradation of MB and oil/water separation for removal of organic pollutants. Appl. Surf. Sci. 2023, 622, 156933 10.1016/j.apsusc.2023.156933.

[ref63] Wang H.; Hu X.; Ke Z.; Du C. Z.; Zheng L.; Wang C.; Yuan Z. Review: Porous Metal Filters and Membranes for Oil-Water Separation. Nanoscale Res. Lett. 2018, 13, 284 10.1186/s11671-018-2693-0. 30209724 PMC6135733

[ref64] Cassie A. B. D.; Baxter S. Wettability of porous surfaces. Trans. Faraday Soc. 1944, 40 (0), 546 10.1039/tf9444000546.

[ref65] Wenzel R. N. Resistance of Solid Surfaces to Wetting by Water. Industrial & Engineering Chemistry 1936, 28 (8), 988–994. 10.1021/ie50320a024.

[ref66] Bormashenko E.; Bormashenko Y.; Stein T.; Whyman G.; Bormashenko E. Why do pigeon feathers repel water? Hydrophobicity of pennae, Cassie-Baxter wetting hypothesis and Cassie-Wenzel capillarity-induced wetting transition. J. Colloid Interface Sci. 2007, 311 (1), 212–216. 10.1016/j.jcis.2007.02.049. 17359990

[ref67] Marmur A. From Hygrophilic to Superhygrophobic: Theoretical Conditions for Making High-Contact-Angle Surfaces from Low-Contact-Angle Materials. Langmuir 2008, 24 (14), 7573–7579. 10.1021/la800304r. 18543997

[ref68] Darmanin T.; Guittard F. Superhydrophobic and superoleophobic properties in nature. Mater. Today 2015, 18 (5), 273–285. 10.1016/j.mattod.2015.01.001.

[ref69] Shayesteh H.; Rahbar-Kelishami A.; Norouzbeigi R. Superhydrophobic/superoleophilic micro/nanostructure nickel particles for oil/water mixture and emulsion separation. Ceram. Int. 2022, 48 (8), 10999–11008. 10.1016/j.ceramint.2021.12.320.

[ref70] Bormashenko E. Liquid marbles: Properties and applications. Curr. Opin. Colloid Interface Sci. 2011, 16 (4), 266–271. 10.1016/j.cocis.2010.12.002.

[ref71] Xu L.; Wu X.; Meng J.; Peng J.; Wen Y.; Zhang X.; Wang S. Papilla-like magnetic particles with hierarchical structure for oil removal from water. Chem. Commun. 2013, 49 (78), 8752–8754. 10.1039/c3cc44003f. 23903298

[ref72] Aussillous P.; Quere D. Liquid marbles. nature 2001, 411, 924–927. 10.1038/35082026. 11418851

[ref73] Lee C. H.; Tiwari B.; Zhang D.; Yap Y. K. Water purification: oil-water separation by nanotechnology and environmental concerns. Environmental Science: Nano 2017, 4 (3), 514–525. 10.1039/C6EN00505E.

[ref74] Zhang J.; Peng K.; Xu Z.-K.; Xiong Y.; Liu J.; Cai C.; Huang X. A comprehensive review on the behavior and evolution of oil droplets during oil/water separation by membranes. Adv. Colloid Interface Sci. 2023, 319, 102971 10.1016/j.cis.2023.102971. 37562248

[ref75] Gupta R. K.; Dunderdale G. J.; England M. W.; Hozumi A. Oil/water separation techniques: a review of recent progresses and future directions. Journal of Materials Chemistry A 2017, 5 (31), 16025–16058. 10.1039/C7TA02070H.

[ref76] Cheryan M.; Rajagopalan N. Membrane processing of oily streams. Wastewater treatment and waste reduction. J. Membr. Sci. 1998, 151 (1), 13–28. 10.1016/S0376-7388(98)00190-2.

[ref77] Hu P.; Ren J.; Ren W.; Sun Y.; Yang H. The feasibility and mechanism of poly-aluminum/titanium silicate composite coagulants for the efficient removal of nano- and micro-sized plastics. Chemical Engineering Journal 2024, 482, 149095 10.1016/j.cej.2024.149095.

[ref78] Wang B.; Handschuh-Wang S.; Shen J.; Zhou X.; Guo Z.; Liu W.; Pumera M.; Zhang L. Small-Scale Robotics with Tailored Wettability. Adv. Mater. 2023, 35 (18), 2205732 10.1002/adma.202205732. 36113864

[ref79] Rius-Ayra O.; Biserova-Tahchieva A.; Sansa-López V.; Llorca-Isern N. Superhydrophobic 304 Stainless Steel Mesh for the Removal of High-Density Polyethylene Microplastics. Langmuir 2022, 38 (18), 5943–5953. 10.1021/acs.langmuir.2c00803. 35465677 PMC9097532

[ref80] Gu G.-h.; Wang H.; Suo J.; Qiu G.-z.; Hao Y. Interfacial interaction of bio-leaching of pyrite mineral. Journal of Central South University 2008, 15, 49–53. 10.1007/s11771-008-0011-1.

[ref81] Li Q.; Wang Q.; Hou J.; Zhang J.; Zhang Y. Aggregating structure in coal water slurry studied by eDLVO theory and fractal dimension. Frontiers in Energy 2023, 17 (2), 306–316. 10.1007/s11708-021-0736-1.

[ref82] Bos R.; van der Mei H. C.; Busscher H. J. Physico-chemistry of initial microbial adhesive interactions-its mechanisms and methods for study. FEMS microbiology reviews 1999, 23 (2), 179–229. 10.1016/S0168-6445(99)00004-2. 10234844

[ref83] Wu Y.; Yang S.; Chai W.; Cao Y. Interaction Forces between Diaspore and Kaolinite in NaOL Solution Probed by EDLVO Theory and AFM Analysis. Minerals 2022, 12 (9), 1123 10.3390/min12091123.

[ref84] Campos A. F. C.; Marinho E. P.; Ferreira M. d. A.; Tourinho F. A.; Paula F. L. d. O.; Depeyrot J. X-DLVO Interactions between nanocolloidal magnetic particles: the quantitative interpretation of the pH-dependent phase diagram of EDL-MF. Brazilian Journal of Physics 2009, 39, 230–235. 10.1590/S0103-97332009000200018.

[ref85] Stöber W.; Fink A.; Bohn E. Controlled growth of monodisperse silica spheres in the micron size range. J. Colloid Interface Sci. 1968, 26 (1), 62–69. 10.1016/0021-9797(68)90272-5.

[ref86] Barale M.; Lefèvre G.; Carrette F.; Catalette H.; Fédoroff M.; Cote G. Effect of the adsorption of lithium and borate species on the zeta potential of particles of cobalt ferrite, nickel ferrite, and magnetite. J. Colloid Interface Sci. 2008, 328 (1), 34–40. 10.1016/j.jcis.2008.09.007. 18834597

[ref87] Chen Z.; Liu J.; Chen C.; Huang Z. Sedimentation of nanoplastics from water with Ca/Al dual flocculants: Characterization, interface reaction, effects of pH and ion ratios. Chemosphere 2020, 252, 126450 10.1016/j.chemosphere.2020.126450. 32222522

[ref88] Sun Q. The physical origin of hydrophobic effects. Chem. Phys. Lett. 2017, 672, 21–25. 10.1016/j.cplett.2017.01.057.

[ref89] Al Harraq A.; Bharti B. Microplastics through the Lens of Colloid Science. ACS Environmental Au 2022, 2 (1), 3–10. 10.1021/acsenvironau.1c00016. 37101760 PMC10125150

[ref90] Zhao J.; Liu F.; Wang Z.; Cao X.; Xing B. Heteroaggregation of Graphene Oxide with Minerals in Aqueous Phase. Environ. Sci. Technol. 2015, 49 (5), 2849–2857. 10.1021/es505605w. 25614925

[ref91] Sun Q.; Su X. W.; Cheng C. B. The dependence of hydrophobic interactions on solute size. Chem. Phys. 2019, 516, 199–205. 10.1016/j.chemphys.2018.09.014.

[ref92] van Oss C. J. The Extended DLVO Theory. Interface Science and Technology 2008, 16, 31–48. 10.1016/S1573-4285(08)00203-2.

[ref93] Santhosh C.; Velmurugan V.; Jacob G.; Jeong S. K.; Grace A. N.; Bhatnagar A. Role of nanomaterials in water treatment applications: A review. Chemical Engineering Journal 2016, 306 (15), 1116–1137. 10.1016/j.cej.2016.08.053.

