# Correction to “Durably Superhydrophobic Magnetic Cobalt Ferrites for Highly Efficient Oil–Water Separation and Fast Microplastic Removal”

**DOI:** 10.1021/acs.langmuir.4c04235

**Published:** 2024-10-31

**Authors:** Anhua Ren, Oriol Rius-Ayra, Min Kang, Nuria Llorca-Isern

(1) Correction of Efficiency Data and Terminology Consistency:

In the “***Results and Discussion***” section, the average microplastic removal efficiency was reported as 98%. However, in the “***Conclusions***” section, the term “separation efficiency” was mistakenly used, and the value was stated as 99%. To ensure consistency in terminology and data, the term “separation efficiency” in the “***Conclusions***” section has been changed to “removal efficiency” and the value has been corrected to 98%, which aligns with the “***Results and Discussion***” section.

Incorrect statement in the original text:

“After ten cycles of plastic removal, the separation efficiency reached 99%.”

Corrected statement:

“After ten cycles of plastic removal, the removal efficiency reached 98%.”

(2) Replacement of Figure 12b:

Figure 12b was inadvertently a duplicate of Figure 11 due to an error during figure preparation. We provide the corrected version of Figure 12b below to accurately represent the data discussed in the manuscript.

**Figure 12:**
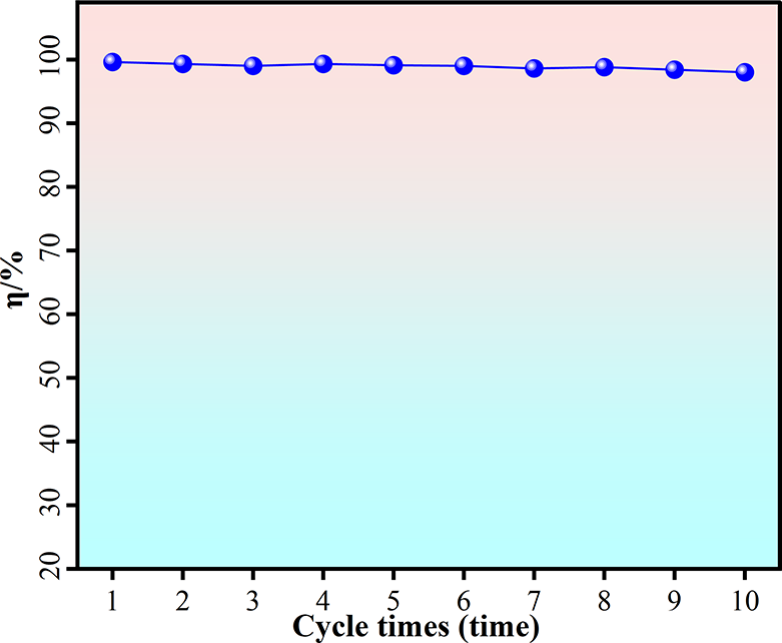
Overview of microplastic removal studies: (b) removal efficiency of microplastics by cobalt ferrites.

We apologize for these errors and any confusion they may have caused. These corrections do not affect the overall conclusions of the study.

